# Down‐regulation of *GIGANTEA*‐*like* genes increases plant growth and salt stress tolerance in poplar

**DOI:** 10.1111/pbi.12628

**Published:** 2016-09-23

**Authors:** Qingbo Ke, Ho Soo Kim, Zhi Wang, Chang Yoon Ji, Jae Cheol Jeong, Haeng‐Soon Lee, Young‐Im Choi, Bingcheng Xu, Xiping Deng, Dae‐Jin Yun, Sang‐Soo Kwak

**Affiliations:** ^1^ Plant Systems Engineering Research Center Korea Research Institute of Bioscience and Biotechnology (KRIBB) Daejeon Korea; ^2^ Department of Green Chemistry and Environmental Biotechnology Korea University of Science and Technology (UST) Daejeon Korea; ^3^ Institute of Soil and Water Conservation Chinese Academy of Science and Ministry of Water Resources Northwest A & F University Shaanxi China; ^4^ Division of Forest Biotechnology Korea Forest Research Institute Suwon Korea; ^5^ Division of Applied Life Science (BK21plus Program), Plant Molecular Biology and Biotechnology Research Center Gyeongsang National University Jinju Korea

**Keywords:** poplar, *Arabidopsis*, *PagGI*, salt tolerance, RNAi, transgenic plants

## Abstract

The flowering time regulator GIGANTEA (GI) connects networks involved in developmental stage transitions and environmental stress responses in *Arabidopsis*. However, little is known about the role of GI in growth, development and responses to environmental challenges in the perennial plant poplar. Here, we identified and functionally characterized three *GI‐like* genes (*PagGIa*,* PagGIb* and *PagGIc)* from poplar (*Populus alba × Populus glandulosa*). *PagGIs* are predominantly nuclear localized and their transcripts are rhythmically expressed, with a peak around zeitgeber time 12 under long‐day conditions. Overexpressing *PagGIs* in wild‐type (WT) *Arabidopsis* induced early flowering and salt sensitivity, while overexpressing *PagGIs* in the *gi‐2* mutant completely or partially rescued its delayed flowering and enhanced salt tolerance phenotypes. Furthermore, the PagGIs‐PagSOS2 complexes inhibited PagSOS2‐regulated phosphorylation of PagSOS1 in the absence of stress, whereas these inhibitions were eliminated due to the degradation of PagGIs under salt stress. Down‐regulation of *PagGIs* by RNA interference led to vigorous growth, higher biomass and enhanced salt stress tolerance in transgenic poplar plants. Taken together, these results indicate that several functions of *Arabidopsis GI* are conserved in its poplar orthologues, and they lay the foundation for developing new approaches to producing salt‐tolerant trees for sustainable development on marginal lands worldwide.

## Introduction

The worldwide population is expected to reach between 9.6 and 12.3 billion by 2100, which presents challenges to energy security, economic growth and environmental protection (Gerland *et al*., 2014). Environmental problems such as global warming, drought and salinity severely limit agricultural and forest productivity (Lobell and Gourdji, 2012; Sivakumar *et al*., 2005). There is an urgent need for plant breeders to develop novel plant varieties with increased growth and tolerance to various environment stresses. Recent studies have revealed that genetic modification of flowering time, or circadian rhythm, represents a useful new strategy for crop and woody plant improvement (Grundy *et al*., 2015; Jung and Müller, 2009; Ni *et al*., 2009).

In the annual plant *Arabidopsis*,* GIGANTEA* (*GI*) was originally identified based on its roles in photoperiodic flowering and circadian clock regulation (Fowler *et al*., 1999; Martin‐Tryon *et al*., 2007). First, the GI‐CONSTANS (CO)‐Flowering Locus T (FT) regulatory module controls flowering time under long‐day conditions (Takada and Goto, 2003). GI and the FLAVIN‐BING, KELCH REPEAT, F‐BOX 1 protein form a complex that controls daytime CO transcription in a light‐dependent manner by degrading a key CO repressor, CYCLING DOF FACTOR 1 (Sawa *et al*., 2007). Second, GI interacts with F‐box protein ZEITLUPE (ZTL) through the amino‐terminal flavin‐binding LIGHT, OXYGEN or VOLTAGE domain of ZTL, which is necessary to sustain a normal circadian period by regulating the proteasome‐dependent degradation of the central circadian oscillator, TIMING OF CAB EXPRESSION 1 (Kim *et al*., 2007). Recent findings indicate that *GI* also mediates responses to environmental stress. Among abiotic stress factors, salinity affects almost all aspects of plant development, including germination, vegetative growth and reproductive development, which threatens crop production on over 800 million hectares, or one‐quarter to one‐third of all agricultural land on earth (Rengasamy, 2010; Zhu, 2002). The salt stress response is controlled by many genes that function through complex genetic regulatory networks. One of the key responses to salt stress is maintaining cellular ion homoeostasis by restricting the accumulation of toxic sodium (Na^+^). The Salt Overly Sensitive (SOS) signalling pathway is a well‐defined signalling pathway required for the control of ion homeostasis (Zhu, 2002). The SOS pathway modulates Na^+^ levels via three known components: calcium‐binding protein SOS3, protein kinase SOS2 and plasma membrane Na^+^/H^+^ antiporter SOS1. Under salt stress conditions, the SOS2–SOS3 complex phosphorylates and activates the transport activity of the SOS1 antiporter (Guo *et al*., 2001; Ji *et al*., 2013; Zhu, 2000). The SOS pathway is functionally conserved in dicot plants *Arabidopsis* and *Brassica nigra*, the monocot plant rice, and the woody plant poplar (Tang *et al*., 2010; Yang *et al*., 2015). The role of *GI* in the salt stress response was recently documented. GI competitively binds to SOS2 kinase and prevents the phosphorylation‐dependent activation of SOS1 under normal conditions. However, in the presence of high salt, GI is degraded by the 26S proteasome. Released SOS2 interacts with SOS3 to form an active SOS2–SOS3 protein kinase complex, which subsequently activates the plasma membrane‐localized Na^+^/H^+^ antiporter SOS1. As a result, sodium ions are exported from the cell and salt tolerance is established (Kim *et al*., 2013a). *GI* is highly conserved in monocot and dicot plants (Song *et al*., 2010). However, the nature of the circadian clock gene *GI* in species other than *Arabidopsis* is much less well understood.

As perennial plants, forest trees are highly valuable, as they provide raw materials, help maintain biodiversity, protect land and water resources, and help mitigate the effects of climate change (Harfouche *et al*., 2011; Lambrou and Laub, 2004). Forest trees have many unique characteristics, such as an extended juvenile phase that can last for decades, critical day length originating from different latitudes, and seasonal changes and cycling between periods of growth and dormancy (Bäurle and Dean, 2006; Jansson and Douglas, 2007). Most poplar cultivars are extremely sensitive to saline soils (Chen *et al*., 2014; Polle and Chen, 2014). Increasing the salt tolerance in crops and woody plants has become a major challenge for modern agriculture. Although much effort has focused on increasing salt tolerance in poplar through conventional breeding and genetic engineering, few studies have successfully produced transgenic poplar plants with enhanced salt tolerance using known abiotic stress‐associated genes from other species (Harfouche *et al*., 2011; Vinocur and Altman, 2005). Recently, transgenic poplar plants overexpressing the poplar *SOS2* gene were generated, which exhibit enhanced tolerance to salt stress (Yang *et al*., 2015). Since *SOS2* is regulated by the photoperiodicity and circadian clock switch *GI* (Kim *et al*., 2013a), unravelling the mechanisms underlying the control of flowering time and its correlation with environmental stress tolerance in trees would greatly accelerate current tree‐breeding programmes.

In this study, we identified three *GI*‐*like* genes (named *PagGIa*,* PagGIb* and *PagGIc*) from poplar (*P. alba × P. glandulosa*) and investigated their roles in circadian rhythm and flowering time control in transgenic *Arabidopsis* plants. In addition, we developed a salt‐tolerant transgenic poplar line by down‐regulating the expression of *PagGIs*, as these genes negatively regulate salt stress tolerance via an SOS‐mediated pathway. This study provides the first evidence that altering the expression of *GI* genes through genetic engineering could be used to develop woody plants with enhanced growth and increased salt tolerance.

## Results

### Cloning and characterization of poplar *GIGANTEA* (*GI)‐like* genes

**Figure 1:**
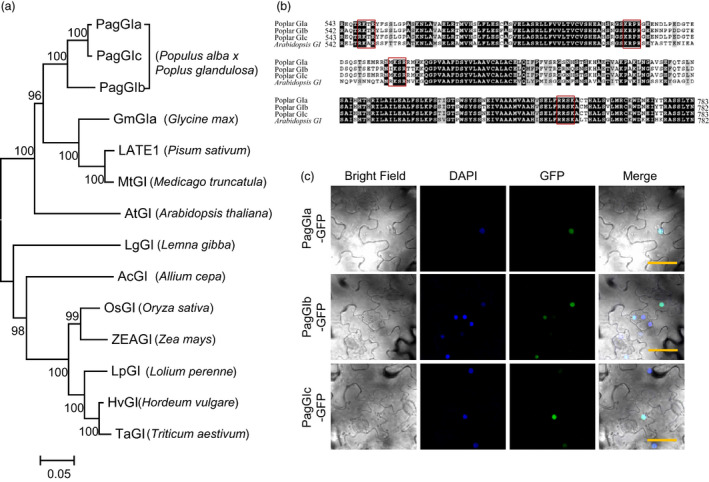
*GIGANTEA*‐*like* Genes in Poplar. (a) Phylogenetic analysis of poplar *GIGANTEA‐like* genes. (b) Comparison of the amino acid sequences of nuclear localization regions of poplar and *Arabidopsis* GI. The alignment was performed using BioEdit and BoxShade server. Identical and similar amino acid residues are shaded in black and grey respectively. The nuclear localization sites are indicated by red boxes. (c) Subcellular localization of PagGIa‐, b‐ and c‐GFP fusion proteins. DAPI (second row) and GFP (third row) fluorescence was observed at 3 days after infiltration. Both fluorescence images are merged in the fourth row (merge). Scale bars represent 50 μM.

We isolated the cDNA fragments of poplar *GI‐like* genes (*PagGIa*,* PagGIb* and *PagGIc)* from *P. alba × P. glandulosa* by homology‐based BLAST searches (DOE JGI, Walnut Creek, CA, USA) against the Joint Genome Institute Phytozome database (www.phytozome.com). Phylogenetic analysis of the deduced plant GI amino acid sequences from 12 species showed that PagGIa, b and c are closely clustered with other dicot *GI* homologues and belong to the same subgroup as AtGI (Figure 1a). These results suggest that *PagGIa*,* b*,* c* and *AtGI* share a conserved role.

Comparison of the deduced amino acid sequences of *AtGI* and the *PagGI* genes revealed that the regions for their nuclear localization are conserved among *Arabidopsis* and poplar *GI*, which contain four clusters of basic amino acids similar to the clusters identified in established bipartite NLSs in other nuclear proteins (Huq *et al*., 2000) (Figure 1b). To investigate the subcellular localization of PagGIa, b and c, we transiently transfected *N. benthamiana* leaves with a *35S*:*PagGIa/b/c*:*GFP* construct. As shown in Figure 1c, GFP fluorescence produced by PagGIa‐, b‐ and c‐GFP fusion proteins overlapped with blue fluorescence, as revealed by 4′,6‐diamidino‐2‐phenylindole (DAPI) staining. These results suggest that PagGIa, b and c predominantly localize to the nucleus and may play a role in transcriptional regulation. GI function in photoperiodic flowering and circadian rhythms have been extensively studied from monocot to dicot plants (Mouradov *et al*., 2002), but have not yet been reported in perennial poplar. To verify whether poplar *PagGIs* are orthologues of *Arabidopsis GI*, we first characterized its original functions in regulation of circadian clock, flowering time.

### *PagGIa*,* b* and *c* are involved in the regulation of circadian rhythm in poplar

**Figure 2:**
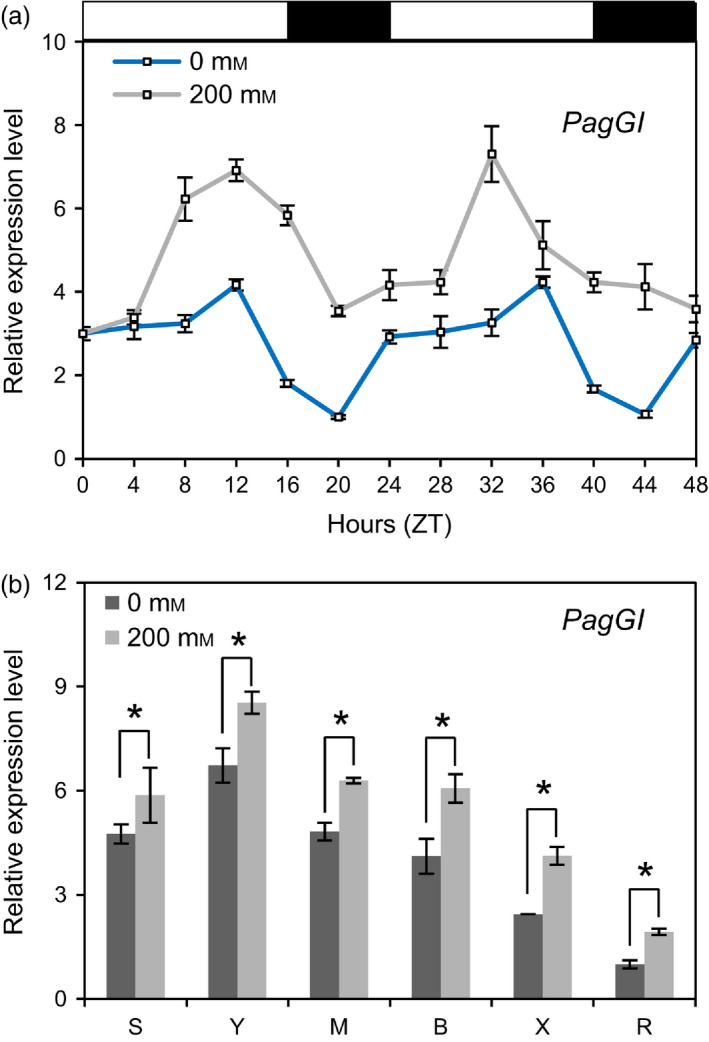
*PagGIa*,* b* and *c* are Involved in the Regulation of Circadian Rhythms. (a) Time course of *PagGI* expression in mature leaves of poplar plants under LDs. (b) Expression levels of *PagGI* in different tissues at ZT12. Total RNA samples were collected every 4 h from 2‐month‐old poplar plants entrained in LDs of 48 h with or without 200 mm NaCl treatments. The mRNA abundance was quantified by quantitative RT‐PCR, which was performed in triplicate with three independently harvested samples. *Actin* expression was used as an internal control. Detected tissues including shoot tip (S), young leaf (Y), mature leaf (M), bark (B), xylem (X) and root (R). White and black bars above the graph indicate day and night periods respectively. Error bars represent SD of three independent experiments. Asterisks indicate significant differences at *P *<* *0.05.

To determine whether *PagGI* mRNA levels fluctuate throughout the day, we examined the expression levels of the *PagGI* genes every 4 hours for 2 days. As shown in Figure 2a, *PagGI* (the sum of *PagGIa*,* b* and *c*) transcripts were diurnally regulated, with a peak around zeitgeber 12 (ZT12) (Zerr *et al*., 1990) in mature leaves. Moreover, *PagGI* mRNAs were most abundant in young leaves among all tissues examined, including shoot tips, young leaves, mature leaves, bark, xylem and roots (Figure 2b). We then determined whether *PagGI* transcript levels were affected by salinity stress. As shown in Figure 2a, *PagGI* mRNA levels were significant up‐regulated throughout the day and in all tissues examined in response to salt stress treatment. We also investigated the transcript levels of *PagGIa*,* b* and *c* separately. Since it is difficult to distinguish between *PagGIa* and *PagGIc* due to their high homology, we listed the expression data for *PagGIa/c*, the sum of *PagGIa* and *PagGIc*. As shown in Figure S1a,b, *PagGIa*/c and *PagGIb* expression was induced by salt stress, and *PagGIb* was expressed at higher levels than *PagGIa/c* throughout the day and in all tissues examined.

### Overexpression of *PagGI*s induces early flowering in *Arabidopsis*

**Figure 3:**
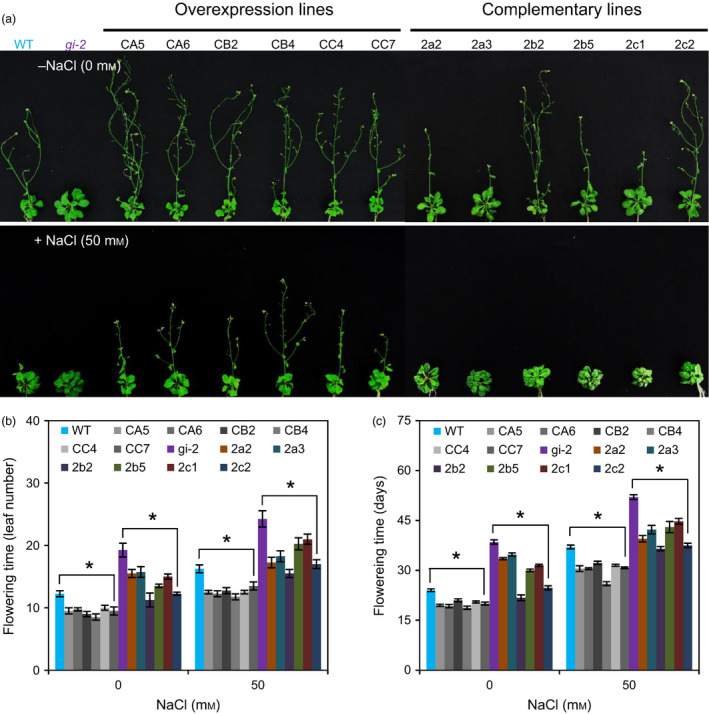
Overexpression of *PagGI* Genes Induces Early Flowering in Transgenic *Arabidopsis* (T3 Generation). (a) Flowering phenotype of *35S*:*PagGIa*/*b*/*c* Col‐0 (overexpression lines) and *35S*:*PagGIa*/*b*/*c gi*‐*2* (complemented lines). Three‐day‐old seedlings were transferred from MS basal medium to MS medium without (0 mm) or with 50 mm NaCl. Plants were grown under LDs and photographed at 5 weeks. Flowering time was scored by counting the number of leaves (rosette and cauline) (b) and measuring the days to bolting (c) when bolted main stems were ~1 cm long. More than 10 plants were measured per data point. Error bars represent SD of three independent experiments. Asterisks indicate significant differences at *P *<* *0.05.

To investigate whether *PagGIa*,* b* and *c* play a functionally conserved role in regulating flowering time, we generated transgenic plants overexpressing *PagGIa*,* b* and *c* in the WT (*35S*:*PagGIa*/*b*/*c* Col‐0) and *gi‐2* mutant (*35S*:*PagGIa*/*b*/*c gi‐2*) backgrounds. We first confirmed the expression levels of *PagGIs* in transgenic *Arabidopsis* by RT‐PCR, immunoblotting analysis, and confocal microscopy (Figure S2a–c). Next, we selected transgenic lines overexpressing the *PagGI* genes (two lines per gene) and subjected them to detailed phenotypic analysis under long‐day conditions (LDs). The *35S*:*PagGIa*/*b*/*c* Col‐0 plants flowered earlier than the WT (Figure 3a–c). *Arabidopsis gi‐2* mutant plants exhibit a strong late‐flowering phenotype (Fowler *et al*., 1999). However, this late‐flowering phenotype was partially or completely rescued in *35S*:*PagGIa*/*b*/*c gi‐2* plants. The *35S*:*PagGIa*/*b*/*c* Col‐*0* and *35S*:*PagGIa*/*b*/*c gi*‐*2* transgenic lines flowered from 15 to 17.8 days and from 19.7 to 38.2 days of seed sowing at the 3/4 (rosette) stage under LDs, whereas WT and *gi‐2* flowered within 19.3 and 41.3 days of seed sowing respectively (Figure 3c). These results suggest that PagGIa, b and c play a role in flowering time regulation in plants.

Salt stress delays the floral transition in *Arabidopsis* (Kim *et al*., 2013a; Li *et al*., 2007). To determine whether *PagGIa*,* b* and *c* transcript levels affect flowering time under salt stress, we observed the flowering time of WT and transgenic *Arabidopsis* plants under salt stress. As shown in Figure 3a–c, salt‐induced late flowering was fully suppressed in *35S*:*PagGIa*/*b*/*c* Col*‐*0 and partially suppressed in *35S*:*PagGIa*/*b*/*c gi‐2* plants, whereas WT displayed a late‐flowering phenotype under salt stress.

### Overexpression of *PagGI* genes confers salt sensitivity in *Arabidopsis*

To determine whether *PagGIa*,* b* and *c* play a functionally conserved role in negatively regulating the salt stress response, we evaluated the roles of *PagGIa*,* b* and *c* in transgenic *Arabidopsis* under salt stress condition. To examine the sensitivity of transgenic *Arabidopsis* seedlings to salt stress, we planted WT, *35S*:*PagGIa*/*b*/*c* Col‐0 and *35S*:*PagGIa*/*b*/*c gi‐2* seeds on MS medium with or without the indicted concentration of NaCl. In the absence of NaCl, all lines exhibited similar seed germination rates. However, in the presence of 100 mm NaCl, *35S*:*PagGIa*/*b*/*c* Col‐0 and *35S*:*PagGIa*/*b*/*c gi*‐*2* seeds exhibited delayed germination compared to WT and *gi*‐*2* seeds respectively (Figure S3a,b). We next examined the root growth phenotype of salt stress‐treated plants. Root elongation was significantly suppressed in *35S*:*PagGIa*/*b*/*c* Col‐0 and *35S*:*PagGIa*/*b*/*c gi*‐*2* compared to WT and *gi*‐*2* plants, respectively, on MS medium containing 125 mm NaCl. The *35S*:*PagGIa*/*b*/*c* Col‐*0* and *35S*:*PagGIa*/*b*/*c gi*‐*2* transgenic plants lost more root fresh weight (78%–85.9% and 47.6%–68%) than WT (59.7%) and *gi‐2* (38.3%) plants respectively (Figure S3c,d).

**Figure 4:**
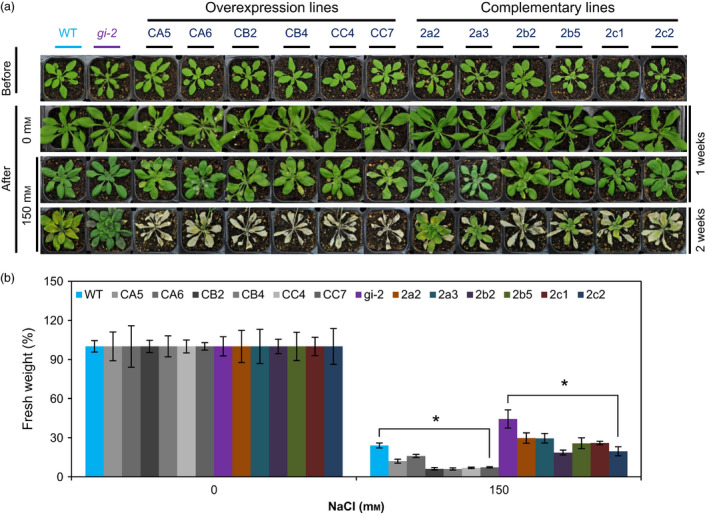
Overexpression of *PagGI* Genes Confers Salt Sensitivity in Transgenic *Arabidopsis*. (a) *35S*:*PagGIa*/*b*/*c* Col‐0 (overexpression lines) and *35S*:*PagGIa*/*b*/*c gi*‐*2* (complemented lines) plants were grown in soil under LDs for 3 weeks (first row, before) and then (after) watered with water (0 mm NaCl) for 1 week (second row) or 150 mm NaCl solution for 1 week (third row) or 2 weeks (fourth row), and (b) relative fresh weights at the end of the treatments (shown in a) were measured. More than 10 plants were measured per data point. Error bars represent SD of three independent experiments. Asterisks indicate significant differences at *P *<* *0.05.

We also evaluated the salt sensitivity of the transgenic *Arabidopsis* plants grown in soil. Compared to WT plants, *35S*:*PagGIa*/*b*/*c* Col*‐0* transgenic lines had reduced tolerance to NaCl‐induced osmotic stress (Figure 4a,b). In addition, the increased salt tolerance of *gi‐2* mutant plants was fully or partially reversed in the *35S*:*PagGIa*/*b*/*c gi‐2* transgenic lines (Figure 4a,b). These results suggest that PagGIa, b and c also function as negative regulators of the salt stress signalling pathway.

### PagGIs inhibit PagSOS2‐dependent phosphorylation of PagSOS1

**Figure 5:**
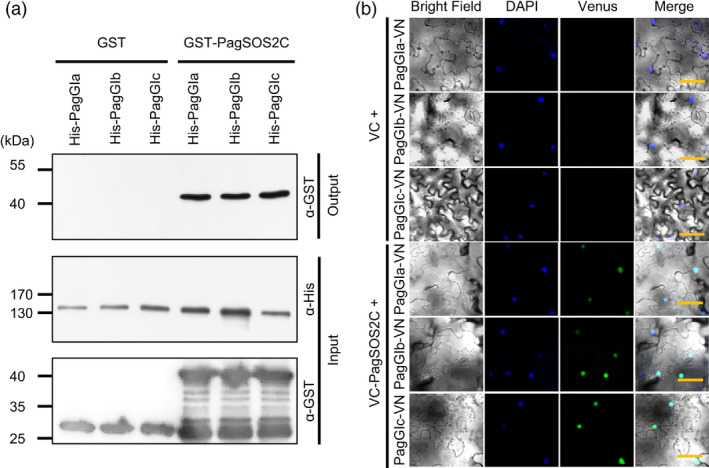
PagGI Proteins Negatively Regulate Salinity Stress Tolerance. (a) His pull‐down assay to examine the association between PagGI proteins and PagSOS2 *in vitro*. For input, the protein extracts were analysed by SDS‐PAGE, followed by western blotting with the indicated antibodies (α‐His, second row; α‐GST, third row). For output, the protein extracts were incubated overnight with His‐agarose beads. After extensive washing, bound proteins were analysed as above with antibodies against GST‐tag (α‐GST, first row). (b) BIFC assay to monitor the interaction between PagGI proteins and PagSOS2 *in vivo*. DAPI (second row) and Venus (third row) fluorescence was observed at 3 days after infiltration. Both fluorescence images are merged in the fourth row (merge). Scale bars represent 50 μM.

We next examined how PagGIa, b, and c participate in the regulation of the salinity stress response. *GI* appears to be involved in salt stress tolerance through the SOS pathway in *Arabidopsis* (Kim *et al*., 2013a). To determine if PagGIs are also involved in the SOS pathway, we isolated genes encoding components of the SOS pathway in poplar, that is, *PagSOS1* and *PagSOS2* (Figure S4 and S5). We next examined the interaction between PagGIs and PagSOS2 via His pull‐down assays *in vitro*. His‐PagGIs and GST‐PagSOS2C fusion proteins were purified as described in Supporting information. His‐PagGIa, ‐b and ‐c were incubated with GST‐PagSOS2C, and the pull‐down extracts were analysed by immunoblotting using the respective anti‐GST antibody. GST‐SOS2 was pulled down with His‐PagGIa, ‐b and ‐c (Figure 5a), suggesting that PagGIa, b and c interact with PagSOS2 *in vitro*. To test the interaction between PagGI proteins and PagSOS2 in plant cells, we used BIFC assays. Cell suspensions of *Agrobacterium tumefaciens* carrying N‐terminal Venus fluorescent protein PagGIa‐, b‐ and c‐VN and C‐terminal Venus fluorescent protein PagSOS2C‐VC constructs were infiltrated into *N. benthamiana* leaves respectively. We detected strong Venus fluorescence in the nucleus when combinations of PagGIa‐, b‐ and c‐VN/PagSOS2C‐VC were used, indicating that PagGI proteins interact with PagSOS2, predominantly in the nucleus (Figure 5b and Figure S6).

**Figure 6:**
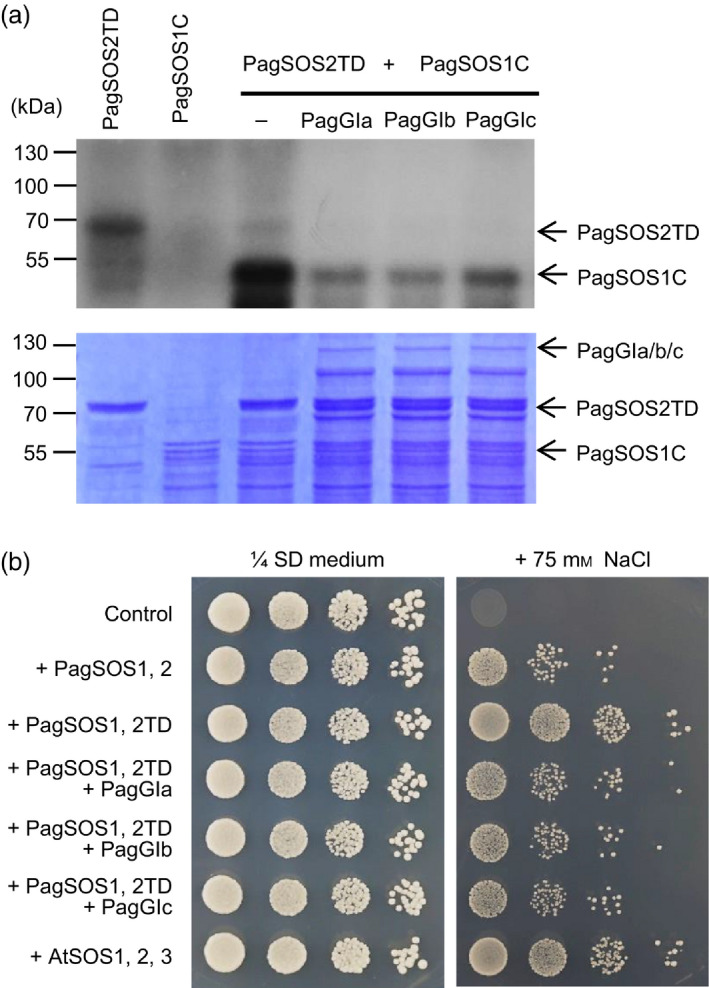
PagGI proteins negatively regulate salinity stress tolerance. (a) PagGIa/b/c inhibit PagSOS2‐mediated PagSOS1 phosphorylation *in vitro*. An *in vitro* kinase assay was performed using purified bacterially GST‐PagSOS1C, GST‐PagSOS2TD and His‐*PagGI* a, b, and c proteins in the indicated combinations. Shown are autoradiogram (top panel) and CBB staining (bottom panel) of a gel containing resolved reactions. (b) PagGIs decrease the NaCl tolerance of yeast expressing the auto‐phosphorylated PagSOS2 (PagSOS2TD)and PagSOS1 ion transporter. Yeast strain AXT3K cells transformed with an empty vector (control) or indicated combination of genes were grown overnight in liquid selective medium. Five microlitres of serial decimal dilutions were spotted onto plates of the same medium or supplemented with 75 mm NaCl. Plates were incubated at 28 °C and photographed after 4 days.

To determine whether the PagGIs‐PagSOS2 complex affects phosphorylation of PagSOS1 by PagSOS2, we performed an *in vitro* kinase assay using a constitutively active form of PagSOS2 (PagSOS2TD), in which Thr168 is changed to Asp. A C‐terminal fragment of SOS1 (PagSOS1C) was used as the substrate. PagSOS2TD showed strong autophosphorylation activity *in vitro*, and it constitutively activated PagSOS1 (Figure S7), whereas when purified recombinant PagGIa, b and c were included in the kinase reaction, the phosphorylation level of PagSOS1 was greatly reduced (Figure 6a). Moreover, we also tested the effects of PagGIs‐PagSOS2 complex on the phosphorylation of PagSOS1 by reconstitution of poplar SOS pathway in yeast cells. As shown in Figure 6b, auto‐phosphorylated PagSOS2 (PagSOS2TD) was sufficient to activate the PagSOS1, however, when coordinate expression of PagSOS2TD and PagGIs, the level of phosphorylated/activated PagSOS1 were greatly reduced in the Yeast *AXT3K* cells (lack the Na^+^ efflux proteins ENA1‐4 and NHA1, and the vacuolar Na^+^/H^+^ antiporter). These results indicate that PagGI proteins negatively regulate salt stress tolerance by inhibiting PagSOS2‐dependent phosphorylation of PagSOS1 via PagGIs‐PagSOS2 interaction.

**Figure 7:**
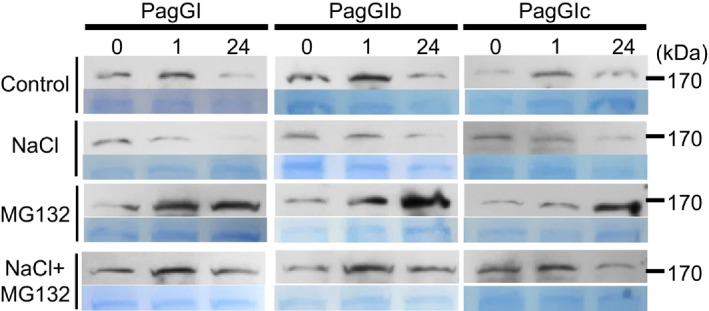
PagGIa, b and c are degraded upon exposure to salt in a proteasome‐dependent manner. Whole 3‐week‐old, soil‐grown *35S*:*PagGIa*/*b*/*c* Col‐0 plants were treated with NaCl (100 mm), MG132 (100 mm) or NaCl plus MG132 at ZT1. PagGIa, b and c protein levels were evaluated after 0, 12 and 24 h treatments via immunoblot analysis with anti‐GFP antibody. Coomassie Brilliant blue (CBB)‐stained blots are shown as a loading control. Molecular weight markers in kDa.

In *Arabidopsis*, AtGI is degraded upon exposure to salt, thus causing the release of PagSOS2, which subsequently regulates the salt stress response (Kim *et al*., 2013a). Therefore, we investigated the protein stability of PagGIa, b and c under salt stress. As shown in Figure 7, PagGIa, b and c protein levels were reduced upon exposure to salt stress, but these decreases were blocked by the proteasome inhibitor MG132, suggesting that PagGIs are unstable proteins that are targeted for 26S proteasome‐dependent proteolysis. Taken together, these results indicate that poplar PagGIa, b and c play a functionally conserved role as negative factors in salt stress tolerance.

### Generation of transgenic poplar with down‐regulated expression of *PagGIs*

**Figure 8:**
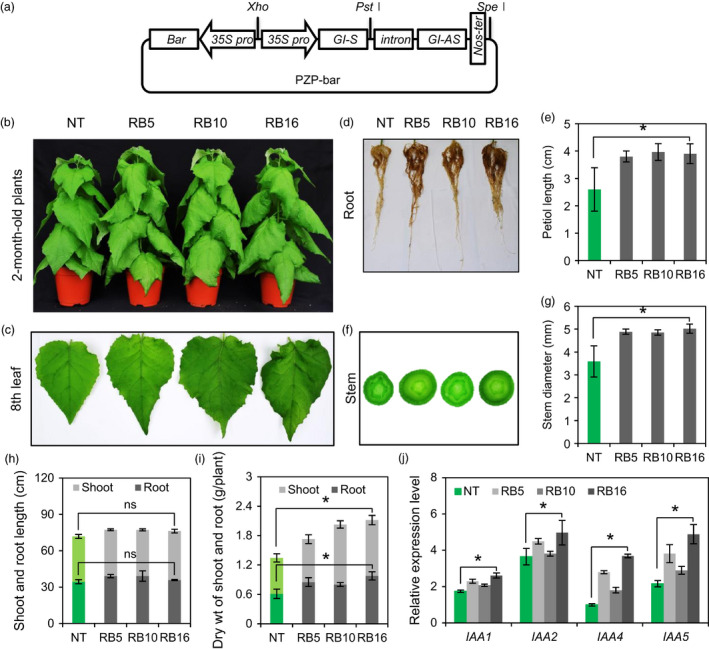
Morphological Phenotypes of RB Poplar Plants. (a) Schematic diagram of the constructs. (b) Plant phenotype of non‐transgenic (NT) and RB plants after 2 months of growth in pots. (c) The 8th leaf, (d) root morphology, (e) petiole length and (f, g) Stem diameter of 2‐month‐old NT and RB plants. Petiole length was determined in the 10th leaves. Stem diameter was measured from the position ~20 cm below the terminal bud. (h) The height and (i) dry weight of shoots and roots of 2‐month‐old poplar plants. (j) Transcript levels of auxin‐responsive *IAA* genes (*IAA1*, *IAA2*, *IAA4* and *IAA5*) were measured by quantitative RT‐PCR in the 10th leaves of 2‐month‐old NT and RB plants. The expression levels of *IAA* genes were normalized to the poplar *actin* gene as an internal control. Data represent three independent experiments. Asterisks and ns indicate significant and nonsignificant differences at *P *<* *0.05 respectively.

The above results encouraged us to generate transgenic poplar plants with improved growth and salt tolerance by down‐regulating *PagGI* genes. An RNA interference (RNAi) construct was produced and introduced into poplar plants (*P. alba × P. glandulosa;* referred to as RB) using *Agrobacterium*‐mediated transformation (Figure 8a). Nine independent RB plants were confirmed by genome PCR analysis with Int F/Nos R‐ and *Bar*‐specific primers, and three lines (RB5, RB16 and RB32) were selected by RT‐PCR and Southern blot analysis for further characterization (Figure S8a–c). To evaluate the morphological phenotypes of non‐transgenic (NT) and RB plants, poplar plants grown on rooting medium (RM) for 1 month were transferred to pots. Two‐month‐old pot‐cultured RB plants exhibited elongated petioles and large leaves with downward curvature (Figure 8b,c). Moreover, RB plants had thick stems and increased biomass (1.29–1.58‐fold higher biomass than NT plants), but no significant difference in height compared to NT plants (Figure 8d–i). Plant viability and developmental processes, including cell division, cell growth and differentiation, are precisely monitored and determined by auxin (Dhonukshe *et al*., 2008). Auxin regulates the expression of numerous auxin‐responsive genes, such as *Aux*/*IAA* genes (Hagen and Guilfoyle, 2002; Kim *et al*., 2011). Interestingly, the transcript levels of early auxin‐response genes including *IAA1*,* IAA2*,* IAA4* and *IAA5* were significantly higher in all RB plants under normal growth conditions compared to the control (Figure 8j). These results indicate that the phenotypes of RB plants are positively correlated with the transcript levels of auxin‐response genes.

### RB plants exhibit increased tolerance to salt stress

**Figure 9:**
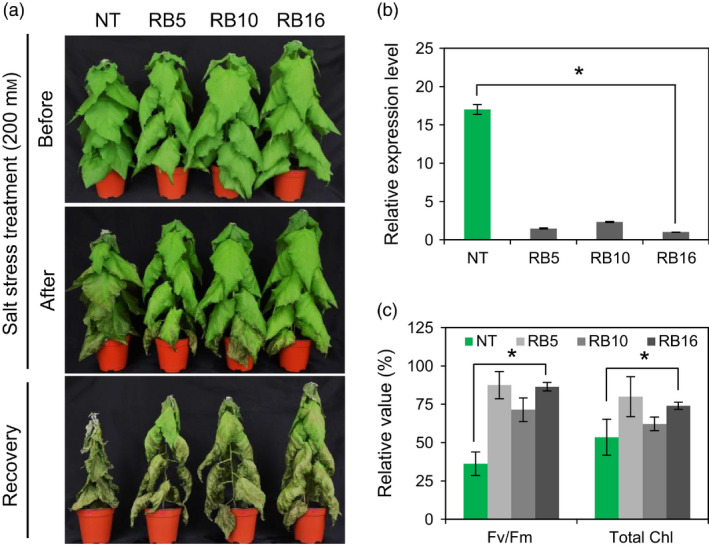
Down‐Regulation of *PagGI* Genes Confers Salt Tolerance in Poplar. (a) Effect of salt stress on RB poplar plants. Two‐month‐old non‐transgenic and RB plants were subjected to 200 mm NaCl treatment for 6 day and recovery for 15 day. (b) Transcript levels of *PagGIs* in three individual RB poplar plants. The 10th leaves from 1‐month‐old plants entrained in LDs were harvested at ZT12. The mRNA abundance was quantified by quantitative RT‐PCR and normalized to the level of poplar *actin* transcript. (c) Fv/Fm and total Chl contents in the leaves (10th) of poplar plants were determined at 4 day after treatment. Data represent means ± SD of three independent experiments. Asterisks indicate significant differences at *P *<* *0.05 respectively.

To evaluate the salt stress tolerance of RB plants, we cultured poplar stem cuttings (NT, RB5, RB10 and RB16) with double nodes in RM tubes supplemented with or without the indicated concentration of NaCl. In the presence of 50 mm NaCl, RB plants exhibited less loss (33.2%–44.1%) of root fresh weight than NT plants (74.7%), whereas very little rooting of NT plants occurred in the presence of 75 mm NaCl after 1 month of treatment (Figure S9a,b). Furthermore, we subjected 2‐month‐old pot‐cultured NT and RB plants of the same health status to 200 mm NaCl for 6 days. NT plants displayed faster wilting and more chlorosis than RB plants under salt stress conditions. The differences in wilting symptoms between NT and RB plants became even more pronounced after normal irrigation was resumed (Figure 9a). As expected, reduced levels of *PagGI* transcripts were detected in RB plants (Figure 9b). RB plants maintained higher photosystem II efficiency (Fv/Fm) and higher levels of chlorophyll compared to NT plants under salt stress, which is consistent with their salt‐resistant phenotypes (Figure 9c). All of these results indicate that down‐regulating *PagGI* genes confers tolerance to salt stress.

## Discussion

### *PagGIs* are functional orthologues of *Arabidopsis GI*

*GIGANTEA* (*GI*) regulates several signalling pathways, but only its role in photoperiodic flowering and circadian rhythms have been extensively studied (Mouradov *et al*., 2002). Poplar differs from annual *Arabidopsis* plant in many ways (Bäurle and Dean, 2006; Jansson and Douglas, 2007). Elucidating how perennial plants adapt their growth and development in response to perceived environmental stress at the molecular level would greatly accelerate current tree‐breeding programmes.

The *GI* gene has undergone several intraspecific gene duplications: soybean has at least four *GI* paralogs (*GmGI 1*α, *GmGI 1*β, *GmGI 2* and *GmGI 3*), and onion has two *GI‐like* genes (*AcGIa* and *AcGIb*) involved in flowering promotion (Taylor *et al*., 2010; Watanabe *et al*., 2011). In this study, we identified three *GI* homologs (paralogs) in poplar. *PagGIa* and *PagGIc* are predicted to be localized to chromosome 5, while *PagGIb* is found on chromosome 2 (data not shown). Although the overall expression profiles of *PagGIa*,* b* and *c* were similar, slight but interesting differences were observed among the three *GI* paralogues. The peak transcript levels were higher for *PagGIb* than the total *PagGIa* and *PagGIc* transcript levels throughout the day and in all investigated tissues (Figure S1a,b). Gene duplication initially generates two identical copies of a gene that are functionally redundant. The original function may be conserved in both genes, providing extra amounts of protein or RNA products, or one gene may evolve freely without much pressure from natural selection until it becomes a non‐functional pseudogene or acquires a novel function (Teichmann and Babu, 2004; Zhang, 2003). Although we detected no significant differences in the physiological functions of these genes, such as the induction of early flowering and salt sensitivity in transgenic *Arabidopsis* (Figure 3a and 4a), the *GI* paralogues appear to be differentially regulated in poplar.

**Figure 10:**
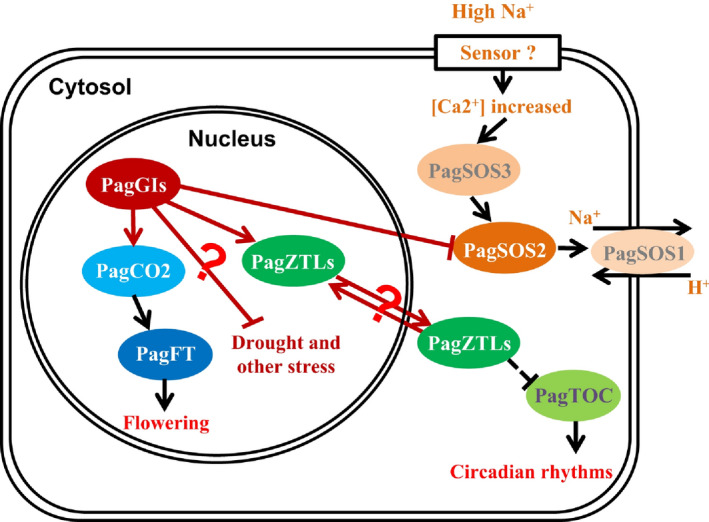
Model of the Roles of *PagGI* Genes in Regulating Circadian Rhythms, Flowering Time and the Salt Stress Response.

Several lines of evidence indicate that *PagGIa*,* b* and *c* are *AtGI* orthologues in poplar (Figure 10). PagGIs regulate circadian rhythms via direct protein–protein interaction with PagZTLs (orthologues of *Arabidopsis* ZTL; Figure S10 and S11), which is necessary to sustain a normal circadian period by controlling the proteasome‐dependent degradation of the central clock protein TIMING OF CAB EXPRESSION 1 (TOC1) (Kim *et al*., 2007, 2013b). Pag*GIs* appear to regulate flowering (at least in part) by influencing PagCO2 (orthologue of *Arabidopsis* CO; Figure S12 and S13), and PagGI‐PagCO‐PagFT (in the photoperiodic pathway) might play a role in regulating both flowering time and the timing of growth cessation (Böhlenius *et al*., 2006). PagGIs negatively regulate salt stress tolerance by caging PagSOS2 to the nucleoplasm and cytoplasm, which might influence the activities of the plasma membrane Na^+^/H^+^ antiporter PagSOS1 (Figure 6). These results provide strong evidence that these three poplar genes are (at least in part) functionally conserved between the herbaceous plant *Arabidopsis* and the woody plant poplar.

### PagGIs play the negative regulatory roles in the salt tolerance response

Among abiotic factors, soil salinity represents an increasing global agricultural and environmental challenge, particularly in irrigated lands (Zhu, 2002). High salt stress disrupts the homoeostasis of water potential and ion distribution. Maintaining ion homoeostasis is critical for plants combating high salinity stress (Zhu, 2000). The initiation of flowering involves components of the circadian clock and is precisely monitored by multiple environmental cues (Andrés and Coupland, 2012; McClung, 2006). A number of components of the circadian clock are transcription factors, which play dual roles in ensuring the functioning of the central oscillator and controlling the rhythmic expression of downstream genes, such as abiotic stress‐responsive genes (Grundy *et al*., 2015). As GIs are large proteins, they possess various functional domains and can participate in multiple signalling pathways. In Arabidopsis, AtGI negatively regulates the salt stress response by caging SOS2 to the nucleoplasm and cytoplasm under normal growth conditions, but is degraded in response to salt stress (Kim *et al*., 2013a). Released SOS2 then interacts with SOS3 to form an active SOS2‐SOS3 protein complex, which regulates the expression and/or activities of various ion transporters, including the plasma membrane Na^+^/H^+^ antiporter SOS1, thereby promoting salt stress tolerance (Guo *et al*., 2004; Zhu, 2000, 2002). The SOS pathway, which functions in the salt stress response, is conserved in poplar: PtSOS1, PtSOS2 and PtSOS3 are functional homologues of their Arabidopsis counterparts, reconstitution of poplar SOS pathway in yeast cells revealed that PtSOS2 and PtSOS3 acted coordinately to activate PtSOS1 (Tang *et al*., 2010). Transgenic poplar plants overexpressing PtSOS2 or PtSOS2TD (constitutively active form of PtSOS2) have improved salt tolerance (Yang *et al*., 2015; Zhou *et al*., 2014). Although the exact mechanism in poplar remains unknown, our findings suggest that *PagGIs* function in the regulation of the salt stress response. Wild‐type *Arabidopsis* plants overexpressing *PagGIa*,* b* and *c* exhibited early flowering and enhanced salt sensitivity, while overexpressing these genes in the *gi‐2 Arabidopsis* mutant rescued the delayed flowering phenotype and increased salt tolerance (Figure 3a, b, Figure 4a, b). His pull‐down assays and BIFC experiments revealed that PagGI proteins interact with PagSOS2 in poplar. PagGI‐PagSOS2 complexes inhibit the PagSOS2‐based phosphorylation of PagSOS1 (Figure 5 and Figure 6a), which subsequently weakens Na^+^/H^+^ ion exchange under normal growth conditions (Figure 6b). PagGIs might play negative roles in salt response either by interfering with the PagSOS2‐regulated phosphorylation of PagSOS1 in cytosol or regulating cytosolic amount of PagSOS2 proteins by sequestering PagSOS2 to the nuclear (Figure 5 and Figure S6). However, it remains to be elucidated. These inhibitions were counteracted by the degradation of PagGI proteins in a proteasome‐dependent manner under salt stress (Figure 7). Down‐regulation of *PagGI* genes confers enhanced salt tolerance in poplar, further confirming that a link exists between salt stress responses and flowering control, and that *PagGI* genes may play pivotal roles in this process.

### Down‐regulation of *PagGIs* by RNA interference led to vigorous growth, higher biomass and enhanced salt stress tolerance in transgenic poplar plants

RNA interference (RNAi) is an effective tool used to manipulate gene expression experimentally and to probe gene function on a genome‐wide scale in both plants and animals (Hannon, 2002). Altering flowering time regulation by overexpressing or suppressing gene activity can be used in crop breeding (Jung and Müller, 2009). Suppressing flowering time‐related gene activity by RNAi has been used to delay bolting and flowering in *Arabidopsis*, rice, and wheat (Curtis *et al*., 2002; Peng *et al*., 2008; Yong *et al*., 2003). The development of salt‐tolerant crops is urgently needed to sustain agricultural production. However, conventional tree‐breeding techniques alone are no longer sufficient to meet the increasing demand for forest products or environmental requirements. Genetic engineering of trees would accelerate the domestication of forest trees (Harfouche *et al*., 2011; Lambrou and Laub, 2004). We previously focused on developing environmental stress‐tolerant poplar plants using known abiotic stress‐related genes from other species (Ke *et al*., 2015; Kim *et al*., 2011). In the current study, we generated transgenic poplar plants with down‐regulated expression of *PagGI* genes (RB plants), and RB plants exhibited increased biomass and tolerance to salt stress (Figure 8a–i). Interestingly, the RB plants displayed auxin‐overproduction morphological phenotypes associated with increased transcript levels of early auxin‐response genes including *IAA1*,* IAA2*,* IAA4* and *IAA5* in all RB plants under normal growth conditions (Figure 8j). Furtermore, GI regulates phytochrome A‐mediated photo‐morphogenesis in *Arabidopsis* (Karina *et al*. 2007). Recent studies have shown that the phytochrome light receptors impose a strong influence on auxin levels in planta, by regulating SUPERROOT 2 (SUR2), a suppressor, and TRYPTOPHAN AMINOTRANSFERASE OF ARABIDOPSIS 1 (TAA1), an enhancer of IAA biosynthesis (Halliday *et al*. 2009). Changed phenotypes of transgenic poplar plants might be achieved by light modulation of auxin signalling systems. However, the relationship between down‐regulation of *GI* genes and increased biomass remains to be elucidated.

Modern‐day plants evolved from their early plant ancestors through long‐term adaption to multiple environmental changes (Niklas and Kutschera, 2010; Tufto, 2000; Zhu, 2002). Perennial woody plants differ from annual plants in several ways and are thought to have evolved a complicated regulatory mechanism in response to environmental cues and seasonal changes (Nicotra *et al*., 2010). In light of the increasingly serious global environmental problems, developing abiotic stress‐tolerant poplar would be quite useful for maintaining biodiversity, moderating climatic conditions and combating desertification (Harfouche *et al*., 2011; Lambrou and Laub, 2004). Our data demonstrate that the link between developmental stage transitions and environmental cues mediated by *GI* is conserved between the model plant *Arabidopsis* and the perennial woody plant poplar. Specifically, we developed transgenic poplar plants with enhanced growth and tolerance to salt stress by down‐regulating *PagGI* genes. Although the performance of these transgenic poplar plants must be further characterized under field conditions, *PagGIs* represent promising candidate genes for use in genetic engineering of salt‐tolerant trees. Therefore, this study provides a new approach for sustainable production of salt‐tolerant forest products on global marginal lands.

## Experimental procedures

### Plant materials and growth conditions

The *Arabidopsis thaliana* ecotype Colombia‐0 (Col‐0) was used as the WT for all experiments. The *gi‐2* mutant (Col‐0 background), which was described previously (Fowler *et al*., 1999), was kindly provided by Dr. Dae‐Jin Yun (Gyeongsang National University, South Korea). *Arabidopsis* seeds were stratified at 4 °C in darkness for 3 day and transferred to a growth chamber at 22 °C under a 16 h light/8 h dark photoperiod (light intensity ~120 μmol/m^2^/s), unless otherwise specified.

A hybrid poplar clone (*P. alba × P. glandulosa*) was used in this study. The plants were sub‐cultured monthly by aseptically transferring shoot apices with double nodes to fresh RM comprising 1× Murashige and Skoog medium (MS, Murashige and Skoog, 1962) containing 0.2 mg/L indole‐3‐butyric acid. After 1 month culture in RM, the rooted plantlets were transplanted to pots and grown in a growth chamber at 25 °C with 60% relative humidity under a light intensity of 150 μmol/m/s and a 16/8 h (light/dark) photoperiod.

### Gene cloning

The poplar *GIGANTEA* (*PagGI*) and *ZEITLUPE* (*PagZTL*) genes were identified by sequence comparison of *Arabidopsis GI* and *ZTL*, respectively, to the *Populous trichocarpa* genomic database. Poplar *CO2* (*PagCO2*) and *SOS2* (*PagSOS2*) were identified based on the cDNA sequences of *PtCO2* and *PtSOS2* respectively. The coding sequences of *PagGIa*,* PagGIb*,* PagGIc*,* PagZTL1*,* PagZTL2*,* PagCO2* and *PagSOS2* were amplified from cDNA from *P. alba × P. glandulosa* leaves with Pfu‐X DNA polymerase (Solgent, Daejeon, Korea) using the primer pairs listed in Table S1. GenBank accession numbers are shown in Table S2.

### Phylogenic analysis

For details, see Supporting information.

### Plasmid construction

For details, see Supporting information.

### RNA preparation and analysis of gene expression

Total RNA was extracted from the indicated plant tissues using a GeneAll Ribospin Plant^™^ kit (GeneAll, Seoul, Korea) according to the manufacturer's instructions. For cDNA production, 2 μg of total RNA was reverse transcribed using an RT‐PCR kit (Enzynomics, Daejeon, Korea). The reaction mixture was diluted to 100 μL with sterilized water, and 2 μL was used for real‐time quantitative RT‐PCR. Gene‐specific primers are listed in Table S1. All quantitative RT‐PCR analysis was performed with a CFX real‐time PCR system and CFX system software (Bio‐Rad, CA) using Ever‐Green 20 fluorescent dye (BioFACT, Daejeon, Korea). The following program was used for PCR: an initial denaturation step for 5 min at 95 °C, followed by 44 cycles of 95 °C for 20 s, 60 °C for 20 s and 72 °C for 20 s. There were at least three biological repeats and three technical repeats per data point.

### *Arabidopsis* and poplar transformation

The PGWB5‐35S:*PagGIa/b/c*‐*GFP* constructs were introduced into *Agrobacterium tumefaciens* strain GV3101 by the freeze‐thaw method (Wise *et al*., 2006) and transferred into WT and *gi‐2* mutant plants by floral dip infiltration (Clough and Bent, 1998). For each gene, two independent transgenic lines (T3 generation), which were homozygous for single‐copy insertions of transgene T‐DNAs in the WT or *gi‐2* mutant background, were used for the experiments.

The indicated construct PZP‐*35S*‐(*GI*‐S)‐Intron‐(*GI*‐AS)‐Nos was introduced into poplar plants by *Agrobacterium tumefaciens* (strain *EHA105*)‐mediated transformation, as described by Choi *et al*. (2005). The transformed calli were selected on callus induction medium (CIM) comprising 1× MS medium containing 1.0 mg/L 2,4‐dichlorophenoxyacetic (2,4‐D), 0.1 mg/L benzylaminopurine (BAP), 0.01 mg/L 1‐naphthylacetic acid (NAA) and the appropriate antibiotics (0.5 mg/L Phosphinothricin [PPT]). PPT‐resistant shoots were regenerated from calli by transferring to shoot induction medium (SIM) comprising 1× Woody Plant Medium (Lloyd and McCown, 1980) containing 1.0 mg/L zeatin, 0.1 mg/L BAP and 0.01 mg/L NAA. Regenerated shoots were transferred to fresh RM for rooting, and the rooted plantlets were used for in‐tube or in‐pot growth assays.

### Salt stress treatment

For details, see Supporting information.

### Transient expression in *N. benthamiana* and confocal microscopy

*Agrobacterium tumefaciens* GV3101 strains carrying the indicated constructs and P19‐silencing plasmid were grown in YEP medium supplemented with the appropriate antibiotics overnight. Cultures were spun down and resuspended in infiltration solution (10 mm MES, 10 mm MgCl_2_ and 100 μM acetosyringone). *Agrobacterium* carrying the indicated construct or combinations of constructs and P19 were co‐infiltrated into 2‐week‐old *N. benthamiana* leaves for subcellular localization analysis. The cultures were grown in infiltration solution to a final OD_600_ = 0.5. After 3 days of growth in a greenhouse at 25 °C under LDs, the infiltrated parts of leaves were cut and immersed in DAPI solution for nuclear staining and subjected to fluorescent signal detection under a Leica TCs SP2 confocal microscope (Leica Microsystems, Heidelberg, Germany) with proper filter sets, as described by Gehl *et al*. (2009).

### Protein expression and purification

For details, see Supporting information.

### Immunoblot analysis

Total protein was extracted from whole plants of 3‐week‐old soil‐grown PagGIa/b/c‐OX plants, which were treated with NaCl (100 mm), MG132 (100 mm) or NaCl plus MG132 at the indicated time point. The extraction solution contained 50 mm Tris‐HCl (pH 7.5), 5 mm EDTA, 5 mm EGTA, 10 mm DTT and one protease inhibitor tablet (Roche, Mannheim, Germany). The extracts were separated by SDS‐PAGE. Immunoblot analysis was performed with anti‐GFP antibody.

### His pull‐down assay

First, 5–10 μg of GST or GST fusion protein (GST‐PagSOS2F or GST‐PagSOS2C) was incubated with 30–50 μg of His fusion protein (His‐PagGIa, His‐PagGIb or His‐PagGIc) in lysis buffer (50 mm Tris‐HCl [pH 7.5], 250 mm NaCl, 5 mm EDTA, 0.1% Tween20, 1 mm DTT and one tablet of protease inhibitor) at 4 °C for 4 h. Next, 50–100 μL of Ni‐NTA agarose was added to the sample, followed by incubation overnight at 4 °C. The beads were washed three times with lysis buffer. Bound proteins were eluted from the beads by boiling in SDS sample buffer and analysed by SDS‐PAGE, followed by staining with Coomassie Brilliant Blue or Western blotting using the appropriate antibodies.

### Kinase assay

For the *in vitro* kinase assay, kinase reactions were performed in 20 μL of kinase buffer (20 mm Tris‐HCl (pH 7.5), 10 mm MgCl_2_, 5 mm EGTA, 100 mm NaCl, and 1 mm DTT) containing 5 μg of fusion protein with 1 μL of [γ‐^32^P] ATP at room temperature for 1 h. The reactions were stopped by adding 4× SDS loading buffer. The phosphorylation of fusion proteins was analysed by autoradiography after separation by 10% SDS‐PAGE.

### Yeast experiments

The *AXT3K* (▵*ena1*::*HIS3*::*ena4*, ▵*nha1*::*LEU2*, ▵*nhx1*::*KanMX4*), which was described previously (Quintero *et al*., 2002), was kindly provided by Dr. Dae‐Jin Yun (Gyeongsang National University, South Korea). The ion uptake experiment was performed as described previously (Ardie *et al*., 2009). Yeast *AXT3K* cells transformed with an empty vector or expressing combinations of PagSOS1, PagSOS2, PagSOS2TD and PagGIa/b/c were incubated in the 1/4 strength SD plate supplemented with or without 75 mm NaCl. Plates were incubated at 28 °C and photographed after 4 days.

### Measurement of flowering time

Flowering time was measured by counting the number of rosette and cauline leaves or days to bolting when floral buds were visible (1 cm long) at the centre of the rosette. Flowering time was measured at least twice with similar results.

### Analysis of photosynthetic activity and chlorophyll contents

Photosynthetic activity in leaves was estimated based on chlorophyll fluorescence determination of photochemical yield (Fv/Fm), which represents the maximal yield of the photochemical reaction in photosystem II (PSII), using a portable chlorophyll fluorescence meter (Handy PEA, Hansatech, England) after 30 min of dark adaption. Chlorophyll contents were measured with a portable chlorophyll meter (SPAD‐502; Konica Minolta, Japan). Total chlorophyll contents after stress treatment were compared with those under normal conditions. Both of these values were detected using the 5th–10th intact, fully expanded leaves (counting from the shoot apical meristem) of individual plants.

### Statistical analysis

Data were statistically analysed with Statistical Package for the Social Sciences (SPSS 12.0, SPSS Inc., Chicago, IL). Means were separated using Duncan's multiple range test at *P *=* *0.05.

## Supporting information


**Figure S1** *PagGI* genes are involved in the regulation of circadian rhythms
**Figure S2** Generation of transgenic *Arabidopsis* plants overexpressing *PagGI* genes
**Figure S3** Sensitivity of transgenic *Arabidopsis* seedlings to salt stress
**Figure S4** *SOS1*‐*like* gene in poplar
**Figure S5** *SOS2*‐*like* gene in poplar
**Figure S6** Nuclear and cytosolic distribution of PagGI proteins
**Figure S7** PagSOS2 phosphorylates PagSOS1 *in vitro*
**Figure S8** Generation of transgenic poplar plants by down‐regulating *PagGI* genes
**Figure S9** Effects of salt stress on NT and RB plants grown under in‐tube conditions
**Figure S10** *ZEITLUPE* (ZTL)‐*like* genes in poplar
**Figure S11** The interaction between PagGI proteins and PagZTL proteins *in vivo*
**Figure S12** *CONSTANS*(*CO*)‐*like* gene in poplar
**Figure S13** The interaction between PagGI proteins and PagCO2 *in vivo*
**Table S1** Gene‐specific primers used in this study.
**Table S2** GenBank accession numbers of genes described in this study.

## References

[pbi12628-bib-0001] Andrés, F. and Coupland, G. (2012) The genetic basis of flowering responses to seasonal cues. Nat. Rev. Genet. 13, 627–639. 22898651 10.1038/nrg3291

[pbi12628-bib-0002] Ardie, S.W. , Xie, L. , Takahashi, R. , Liu, S. and Takano, T. (2009) Cloning of a high‐affinity K^+^ transporter gene PutHKT2; 1 from Puccinellia tenuiflora and its functional comparison with OsHKT2; 1 from rice in yeast and *Arabidopsis* . J. Exp. Bot. 184, 1–2. 10.1093/jxb/erp184 PMC2724696 19528529

[pbi12628-bib-0003] Bäurle, I. and Dean, C. (2006) The timing of developmental transitions in plants. Cell, 125, 655–664. 16713560 10.1016/j.cell.2006.05.005

[pbi12628-bib-0004] Böhlenius, H. , Huang, T. , Charbonnel‐Campaa, L. , Brunner, A.M. , Jansson, S. , Strauss, S.H. and Nilsson, O. (2006) CO/FT regulatory module controls timing of flowering and seasonal growth cessation in trees. Science, 312, 1040–1043. 16675663 10.1126/science.1126038

[pbi12628-bib-0005] Chen, S. , Hawighorst, P. , Sun, J. and Polle, A. (2014) Salt tolerance in *Populus*: significance of stress signaling networks, mycorrhization, and soil amendments for cellular and whole‐plant nutrition. Environ. Exp. Bot. 107, 113–124.

[pbi12628-bib-0006] Choi, Y.I. , Noh, E.W. , Lee, H.S. , Han, M.S. , Lee, J.S. and Choi, K.S. (2005) An efficient and novel plant selectable marker based on organomercurial resistance. J. Plant Biol. 48, 351–355.

[pbi12628-bib-0007] Clough, S.J. and Bent, A.F. (1998) Floral dip: a simplified method for *Agrobacterium*‐mediated transformation of *Arabidopsis thaliana* . Plant J. 16, 735–743. 10069079 10.1046/j.1365-313x.1998.00343.x

[pbi12628-bib-0008] Curtis, I.S. , Nam, H.G. , Yun, J.Y. and Seo, K.H. (2002) Expression of an antisense *GIGANTEA* (*GI*) gene fragment in transgenic radish causes delayed bolting and flowering. Transgenic Res. 11, 249–256. 12113457 10.1023/a:1015655606996

[pbi12628-bib-0009] Dhonukshe, P. , Tanaka, H. , Goh, T. , Ebine, K. , Mähönen, A.P. , Prasad, K. , Blilou, I. *et al*. (2008) Generation of cell polarity in plants links endocytosis, auxin distribution and cell fate decisions. Nature, 456, 962–966. 18953331 10.1038/nature07409 PMC2692841

[pbi12628-bib-0010] Fowler, S. , Lee, K. , Onouchi, H. , Samach, A. , Richardson, K. , Morris, B. , Coupland, G. *et al*. (1999) *GIGANTEA*: a circadian clock‐controlled gene that regulates photoperiodic flowering in *Arabidopsis* and encodes a protein with several possible membrane‐spanning domains. EMBO J. 18, 4679–4688. 10469647 10.1093/emboj/18.17.4679 PMC1171541

[pbi12628-bib-0011] Gehl, C. , Waadt, R. , Kudla, J. , Mendel, R.R. and Hänsch, R. (2009) New GATEWAY vectors for high throughput analyses of protein‐protein interactions by bimolecular fluorescence complementation. Mol. Plant, 2, 1051–1058. 19825679 10.1093/mp/ssp040

[pbi12628-bib-0012] Gerland, P. , Raftery, A.E. , Ševčíková, H. , Li, N. , Gu, D. , Spoorenberg, T. , Alkema, L. *et al*. (2014) World population stabilization unlikely this century. Science, 346, 234–237. 25301627 10.1126/science.1257469 PMC4230924

[pbi12628-bib-0013] Grundy, J. , Stoker, C. and Carré, I.A. (2015) Circadian regulation of abiotic stress tolerance in plants. Front Plant Sci. 6, 648. 26379680 10.3389/fpls.2015.00648 PMC4550785

[pbi12628-bib-0014] Guo, Y. , Halfter, U. , Ishitani, M. and Zhu, J.K. (2001) Molecular characterization of functional domains in the protein kinase SOS2 that is required for plant salt tolerance. Plant Cell, 13, 1383–1400. 11402167 10.1105/tpc.13.6.1383 PMC135579

[pbi12628-bib-0015] Guo, Y. , Qiu, Q.S. , Quintero, F.J. , Pardo, J.M. , Ohta, M. , Zhang, C. , Schumaker, K.S. *et al*. (2004) Transgenic evaluation of activated mutant alleles of *SOS2* reveals a critical requirement for its kinase activity and C‐terminal regulatory domain for salt tolerance in *Arabidopsis thaliana* . Plant Cell, 16, 435–449. 14742879 10.1105/tpc.019174 PMC341915

[pbi12628-bib-0016] Hagen, G. and Guilfoyle, T. (2002) Auxin‐responsive gene expression: genes, promoters and regulatory factors. Plant Mol. Biol. 49, 373–385. 12036261

[pbi12628-bib-0062] Halliday, K.J. , Martínez‐García, J.F. , Josse, E.M. (2009) Integration of light and auxin signaling. CSH Perspect Biol. 1, a001586. 10.1101/cshperspect.a001586 PMC2882117 20457562

[pbi12628-bib-0017] Hannon, G.J. (2002) RNA interference. Nature, 418, 244–251. 12110901 10.1038/418244a

[pbi12628-bib-0018] Harfouche, A. , Meilan, R. and Altman, A. (2011) Tree genetic engineering and applications to sustainable forestry and biomass production. Trends Biotechnol. 29, 9–17. 20970211 10.1016/j.tibtech.2010.09.003

[pbi12628-bib-0019] Huq, E. , Tepperman, J.M. and Quail, P.H. (2000) GIGANTEA is a nuclear protein involved in phytochrome signaling in *Arabidopsis* . Proc. Natl Acad. Sci. USA, 97, 9789–9794. 10920210 10.1073/pnas.170283997 PMC16943

[pbi12628-bib-0021] Jansson, S. and Douglas, C.J. (2007) *Populus*: a model system for plant biology. Annu. Rev. Plant Biol. 58, 435–458. 17280524 10.1146/annurev.arplant.58.032806.103956

[pbi12628-bib-0022] Ji, H. , Pardo, J.M. , Batelli, G. , Van Oosten, M.J. , Bressan, R.A. and Li, X. (2013) The Salt Overly Sensitive (SOS) pathway: established and emerging roles. Mol. Plant, 6, 275–286. 23355543 10.1093/mp/sst017

[pbi12628-bib-0023] Jung, C. and Müller, A.E. (2009) Flowering time control and applications in plant breeding. Trends Plant Sci. 14, 563–573. 19716745 10.1016/j.tplants.2009.07.005

[pbi12628-bib-0024] Ke, Q. , Wang, Z. , Ji, C.Y. , Jeong, J.C. , Lee, H.S. , Li, H. , Xu, B. *et al*. (2015) Transgenic poplar expressing *Arabidopsis YUCCA6* exhibits auxin‐overproduction phenotypes and increased tolerance to abiotic stress. Plant Physiol. Biochem. 94, 19–27. 25980973 10.1016/j.plaphy.2015.05.003

[pbi12628-bib-0025] Kim, W.Y. , Fujiwara, S. , Suh, S.S. , Kim, J. , Kim, Y. , Han, L. , David, K. *et al*. (2007) ZEITLUPE is a circadian photoreceptor stabilized by GIGANTEA in blue light. Nature, 449, 356–360. 17704763 10.1038/nature06132

[pbi12628-bib-0026] Kim, Y.H. , Kim, M.D. , Choi, Y.I. , Park, S.C. , Yun, D.J. , Noh, E.W. , Lee, H.S. *et al*. (2011) Transgenic poplar expressing *Arabidopsis NDPK2* enhances growth as well as oxidative stress tolerance. Plant Biotechnol. J. 9, 334–347. 20649941 10.1111/j.1467-7652.2010.00551.x

[pbi12628-bib-0027] Kim, W.Y. , Ali, Z. , Park, H.J. , Park, S.J. , Cha, J.Y. , Perez‐Hormaeche, J. , Quintero, F.J. *et al*. (2013a) Release of SOS2 kinase from sequestration with GIGANTEA determines salt tolerance in *Arabidopsis* . Nat. Commun. 4, 1352. 23322040 10.1038/ncomms2357

[pbi12628-bib-0028] Kim, J. , Geng, R. , Gallenstein, R.A. and Somers, D.E. (2013b) The F‐box protein ZEITLUPE controls stability and nucleocytoplasmic partitioning of GIGANTEA. Development, 140, 4060–4069. 24004949 10.1242/dev.096651 PMC3775418

[pbi12628-bib-0029] Lambrou, Y. , Laub, R. (2004) Gender perspectives on the conventions on biodiversity, climate change and desertification. United Nations, FAO: Gender and Population Division.

[pbi12628-bib-0030] Li, K. , Wang, Y. , Han, C. , Zhang, W. , Jia, H. and Li, X. (2007) GA signaling and CO/FT regulatory module mediate salt‐induced late flowering in *Arabidopsis thaliana* . Plant Growth Regul. 53, 195–206.

[pbi12628-bib-0031] Lloyd, G. and McCown, B. (1980) Commercially‐feasible micropropagation of mountain laurel, Kalmia latifolia, by use of shoot‐tip culture. Comb. Proc. Int. Plant Prop. Soc. 30, 421–427.

[pbi12628-bib-0032] Lobell, D.B. and Gourdji, S.M. (2012) The influence of climate change on global crop productivity. Plant Physiol. 160, 1686–1697. 23054565 10.1104/pp.112.208298 PMC3510102

[pbi12628-bib-0033] Martin‐Tryon, E.L. , Kreps, J.A. and Harmer, S.L. (2007) *GIGANTEA* acts in blue light signaling and has biochemically separable roles in circadian clock and flowering time regulation. Plant Physiol. 143, 473–486. 17098855 10.1104/pp.106.088757 PMC1761957

[pbi12628-bib-0034] McClung, C.R. (2006) Plant circadian rhythms. Plant Cell, 18, 792–803. 16595397 10.1105/tpc.106.040980 PMC1425852

[pbi12628-bib-0036] Mouradov, A. , Cremer, F. and Coupland, G. (2002) Control of flowering time interacting pathways as a basis for diversity. Plant Cell, 14, S111–S130. 12045273 10.1105/tpc.001362 PMC151251

[pbi12628-bib-0037] Murashige, T. and Skoog, F. (1962) A revised medium for rapid growth and bio assays with tobacco tissue cultures. Physiol. Plant, 15, 473–497.

[pbi12628-bib-0038] Ni, Z. , Kim, E.D. , Ha, M. , Lackey, E. , Liu, J. , Zhang, Y. , Sun, Q. *et al*. (2009) Altered circadian rhythms regulate growth vigour in hybrids and allopolyploids. Nature, 457, 327–331. 19029881 10.1038/nature07523 PMC2679702

[pbi12628-bib-0039] Nicotra, A.B. , Atkin, O.K. , Bonser, S.P. , Davidson, A.M. , Finnegan, E. , Mathesius, U. , Poot, P. *et al*. (2010) Plant phenotypic plasticity in a changing climate. Trends Plant Sci. 15, 684–692. 20970368 10.1016/j.tplants.2010.09.008

[pbi12628-bib-0040] Niklas, K.J. and Kutschera, U. (2010) The evolution of the land plant life cycle. New Phytol. 185, 27–41. 19863728 10.1111/j.1469-8137.2009.03054.x

[pbi12628-bib-0041] Peng, L.T. , Shi, Z.Y. , Li, L. , Shen, G.Z. and Zhang, J.L. (2008) Overexpression of transcription factor OsLFL1 delays flowering time in *Oryza sativa* . J. Plant Physiol. 165, 876–885. 17913295 10.1016/j.jplph.2007.07.010

[pbi12628-bib-0042] Polle, A. and Chen, S. (2014) On the salty side of life: molecular, physiological and anatomical adaptation and acclimation of trees to extreme habitats. Plant, Cell Environ. 38, 1794–1816. 25159181 10.1111/pce.12440

[pbi12628-bib-0063] Quintero, F.J. , Ohta, M. , Shi, H. , Zhu, J.K. , Pardo, J.M. , (2002) Reconstitution in yeast of the Arabidopsis SOS signaling pathway for Na^+^ homeostasis. Proc. Natl Acad. Sci. USA, 99, 9061–9066. 12070350 10.1073/pnas.132092099 PMC124423

[pbi12628-bib-0043] Rengasamy, P. (2010) Soil processes affecting crop production in salt‐affected soils. Funct. Plant Biol. 37, 613–620.

[pbi12628-bib-0045] Sivakumar, M. , Das, H. and Brunini, O. (2005) Impacts of present and future climate variability and change on agriculture and forestry in the arid and semi‐arid tropics. Clim. Change, 70, 31–72.

[pbi12628-bib-0046] Song, Y.H. , Ito, S. and Imaizumi, T. (2010) Similarities in the circadian clock and photoperiodism in plants. Curr. Opin. Plant Biol. 13, 594–603. 20620097 10.1016/j.pbi.2010.05.004 PMC2965781

[pbi12628-bib-0061] Takada, S. , Goto, K. (2003) TERMINAL FLOWER2, an *Arabidopsis* homolog of HETEROCHROMATIN PROTEIN1, counteracts the activation of *FLOWERING LOCUS T* by CONSTANS in the vascular tissues of leaves to regulate flowering time. Plant Cell, 15, 2856–2865. 14630968 10.1105/tpc.016345 PMC282816

[pbi12628-bib-0047] Tang, R.J. , Liu, H. , Bao, Y. , Lv, Q.D. , Yang, L. and Zhang, H.X. (2010) The woody plant poplar has a functionally conserved salt overly sensitive pathway in response to salinity stress. Plant Mol. Biol. 74, 367–380. 20803312 10.1007/s11103-010-9680-x

[pbi12628-bib-0048] Taylor, A. , Massiah, A.J. and Thomas, B. (2010) Conservation of *Arabidopsis thaliana* photoperiodic flowering time genes in onion (*Allium cepa L.)* . Plant Cell Physiol. 51, 1638–1647. 20709686 10.1093/pcp/pcq120

[pbi12628-bib-0049] Teichmann, S.A. and Babu, M.M. (2004) Gene regulatory network growth by duplication. Nat. Genet. 36, 492–496. 15107850 10.1038/ng1340

[pbi12628-bib-0050] Tufto, J. (2000) The evolution of plasticity and nonplastic spatial and temporal adaptations in the presence of imperfect environmental cues. Am. Nat. 156, 121–130. 10856196 10.1086/303381

[pbi12628-bib-0051] Vinocur, B. and Altman, A. (2005) Recent advances in engineering plant tolerance to abiotic stress: achievements and limitations. Curr. Opin. Biotechnol. 16, 123–132. 15831376 10.1016/j.copbio.2005.02.001

[pbi12628-bib-0052] Watanabe, S. , Xia, Z. , Hideshima, R. , Tsubokura, Y. , Sato, S. , Yamanaka, N. , Takahashi, R. *et al*. (2011) A map‐based cloning strategy employing a residual heterozygous line reveals that the *GIGANTEA* gene is involved in soybean maturity and flowering. Genetics, 188, 395–407. 21406680 10.1534/genetics.110.125062 PMC3122305

[pbi12628-bib-0053] Wise, A.A. , Liu, Z. and Binns, A.N. (2006) Three methods for the introduction of foreign DNA into *Agrobacterium* . Agrobacterium Protoc. 343, 43–54. 10.1385/1-59745-130-4:43 16988332

[pbi12628-bib-0054] Yang, Y. , Tang, R.J. , Jiang, C.M. , Li, B. , Kang, T. , Liu, H. , Zhao, N. *et al*. (2015) Overexpression of the *PtSOS2* gene improves tolerance to salt stress in transgenic poplar plants. Plant Biotechnol. J. 13, 962–973. 25641517 10.1111/pbi.12335

[pbi12628-bib-0055] Yong, W.D. , Xu, Y.Y. , Xu, W.Z. , Wang, X. , Li, N. , Wu, J.S. , Liang, T.B. *et al*. (2003) Vernalization‐induced flowering in wheat is mediated by a lectin‐like gene *VER2* . Planta, 217, 261–270. 12783334 10.1007/s00425-003-0994-7

[pbi12628-bib-0056] Zerr, D. , Hall, J. , Rosbash, M. and Siwicki, K. (1990) Circadian fluctuations of period protein immunoreactivity in the CNS and the visual system of Drosophila. J. Neurosci. 10, 2749–2762. 2117644 10.1523/JNEUROSCI.10-08-02749.1990 PMC6570283

[pbi12628-bib-0057] Zhang, J. (2003) Evolution by gene duplication: an update. Trends Ecol. Evol. 18, 292–298.

[pbi12628-bib-0058] Zhou, J. , Wang, J. , Bi, Y. , Wang, L. , Tang, L.Z. , Yu, X. , Ohtani, M. *et al*. (2014) Overexpression of PtSOS2 enhances salt tolerance in transgenic poplars. Plant Mol. Biol. Rep. 32, 185–197. 10.1007/s11105-013-0640-x PMC3893482 24465084

[pbi12628-bib-0059] Zhu, J.K. (2000) Genetic analysis of plant salt tolerance using *Arabidopsis* . Plant Physiol. 124, 941–948. 11080272 10.1104/pp.124.3.941 PMC1539290

[pbi12628-bib-0060] Zhu, J.K. (2002) Salt and drought stress signal transduction in plants. Annu. Rev. Plant Biol. 53, 247–273. 12221975 10.1146/annurev.arplant.53.091401.143329 PMC3128348

