# Supplementary material for: Down‐regulation of GIGANTEA ‐like genes increases plant growth and salt stress tolerance in poplar
Source: Plant Biotechnol J. 2016 Sep 23;15(3):331–43. doi: 10.1111/pbi.12628 (PMC5316923; doi:10.1111/pbi.12628)
Supplement: Supplementary file 1 — Figure S1 PagGI genes are involved in the regulation of circadian rhythms Figure S2 Generation of transgenic Arabidopsis plants overexpressing PagGI genes Figure S3 Sensitivity of transgenic Arabidopsis seedlings to salt stress Figure S4 SOS1‐like gene in poplar Figure S5 SOS2‐like gene in poplar Figure S6 Nuclear and cytosolic distribution of PagGI proteins Figure S7 PagSOS2 phosphorylates PagSOS1 in vitro Figure S8 Generation of transgenic poplar plants by down‐regulating PagGI genes Figure S9 Effects of salt stress on NT and RB plants grown under in‐tube conditions Figure S10 ZEITLUPE (ZTL)‐like genes in poplar Figure S11 The interaction between PagGI proteins and PagZTL proteins in vivo Figure S12 CONSTANS(CO)‐like gene in poplar Figure S13 The interaction between PagGI proteins and PagCO2 in vivo Table S1 Gene‐specific primers used in this study. Table S2 GenBank accession numbers of genes described in this study. [file PBI-15-331-s001.docx]

**Supporting information**

**Plasmid construction**

The attB site-containing stop-codon-less ORF sequences of *PagGIa*, *PagGIb*, *PagGIc*, *PagZTL1*, *PagZTL2*, *PagCO2*, full-length *PagSOS2* (*PagSOS2F*, 1–444 aa), and *PagSOS2* C-terminal fragment (*PagSOS2C*, 310–445 aa), a constitutively active form of *PagSOS2* (*PagSOS2TD*, which was generated by site-directed mutagenesis of Thr168 into Asp using a QuikChange II XL Site-Directed Mutagenesis Kit [Agilent, CA, USA]), full-length *PagSOS1* (*PagSOS1F*, 1–1146 aa), and *PagSOS1* C-terminal fragment (*PagSOS1C*, 885–1146 aa) were amplified with the indicated primers shown in Supplemental Table S1 to generate entry vectors based on pDONR207 using BP Clonase (Invitrogen, CA, USA).

For subcellular localization analysis, *PagGIa*-, *PagGIb*-, *PagGIc*-, *PagZTL1*-, *PagZTL2*-, *PagCO2*-, *PagSOS2F*-, *PagSOS2C*-, *PagSOS1C*, and *PagSOS1C*-PDONR207 were cloned into destination vector PGWB5 using LR Clonase (Invitrogen, CA, USA) to create the corresponding GFP fusion proteins.

For bimolecular fluorescence complementation, PagGIa, PagGIb, and PagGIc were transferred from the entry vectors to destination vector pDEST-VYNE(R)^GW^ (containing the N-terminal fragment of Venus fluorescent protein), respectively, using LR Clonase, while *PagZTL1*, *PagZTL2*, *PagCO2*, *PagSOS2F*, and *PagSOS2C* were transferred from the entry vectors to destination vector pDEST-VYCE(R)^GW^ (containing the C-terminal fragment of Venus fluorescent protein), respectively, using LR Clonase.

For the pull-down and kinase assays, *PagGIa*, *PagGIb* and *PagGIc* were transferred from the entry vectors to destination vector pDEST17 to generate His-tag fusion proteins, respectively, using LR Clonase, while *PagSOS2F*, *PagSOS2C*, *PagSOS2TD*, and *PagSOS1C* were transferred from the entry vectors to destination vector pDEST15 to generate GST-tag fusion proteins, respectively, using LR Clonase.

For yeast experiments, the full-length *PagSOS1* cds was subcloned as an *Xho* I fragment, and then the *Xho* I fragment was infused into pDR195 under the control of the pPMA1 using an In-fusion HD cloning kit (Takara, Dalian, China). The full-length *PagSOS2/PagSOS2TD* cds were subcloned as an *Sma* I-*Eco*R I fragment into pBEVY-L under the control of the ADH1. To coordinate expression of *PagSOS2/PagSOS2TD* and *PagGIa/b/c* from a single plasmid, the *Bam*H I-*Sal* I fragment were infused into pBEVY-L-ADH1- *PagSOS2/PagSOS2TD* using an In-fusion HD cloning kit (Takara, Dalian, China).

To construct the *PagGIs* RNAi vector, the dsRNA construct was produced, which contained the cauliflower mosaic virus (CaMV) 35S promoter, a sense fragment of *PagGI* cDNA (GI-S, a region showing high similarity to *PagGIa*, *PagGIb*, and *PagGIc*, but not to other genes, which was identified using BLOCK-iT^TM^ RNAi Designer [Invitrogen, CA, USA]), a 120-nucleotide intron of *A. thaliana* *RTM1*, the *PagGI* fragment in antisense orientation (GI-AS), and a *Nos* terminator. All fragments were obtained using Pfu-X DNA polymerase (Solgent, Daejeon, Korea) with the indicated primer pairs listed in Supplemental Table S1. The 35S-(GI-S) and Intron-(GI-AS)-Nos constructs were first obtained using an In-fusion HD cloning kit (Takara, Dalian, China). PZP-35S-(GI-S)-Intron-(GI-AS)-Nos vector was constructed via two steps of ligation using the 35S fragment with an *Xho*I site in its 5’ terminus, a *Sac*I site in the 3’ terminus of GI-S, and a 5’ terminal intron fragment and *Spe*I site at the 3’ terminus of the Nos fragment. All cloning reactions were performed according to the manufacturer’s instructions.

**Phylogenic analysis**

Sequences of *GI* and *ZTL* family members were identified by BLAST searches in GenBank. The published sequences were collated and converted to predicted amino acid sequences using BioEdit. Alignments were carried out using BioEdit and BoxShade server. Phylogenetic trees were constructed using Molecular Evolutionary Genetics Analysis Version 6. Neighbor-joining phylogenetic trees were created with 1,000 bootstrap replicates.

**Protein expression, purification and fractionation**

For protein expression, *Escherichia coli* BL21 (DE3) cells containing GST fusion proteins (GST-PagSOS2F and GST-PagSOS2C) or 6*×* His fusion proteins (His-PagGIa, His-PagGIb, and His-PagGIc) were induced by treatment with 0.5 mM isopropyl β-D-1-thiogalactopyranoside (IPTG) overnight at 15°C (for His-PagGIa, His-PagGIb, and His-PagGIc), or 0.5 mM IPTG for 5 h at 30°C (for GST-PagSOS2F, GST-PagSOS2C, GST-PagSOS2TD, and GST-PagSOS1C). The cells were disrupted by sonication and shaken rapidly on ice for 1 h after the addition of 1% Triton X-100. The samples were then centrifuged and the supernatant was transferred to a new tube. The supernatants were incubated overnight with prewashed GST beads (GE Healthcare, Uppsala, Sweden) or Ni-NTA beads (Qiagen, Hilden, Germany) with gentle rotation at 4°C. The beads were extensively washed, and the fusion protein was eluted with 10 mM glutathione (Sigma-Aldrich, MI, USA) for GST-tag fusion proteins, or 250 mM imidazole (Fluka, Buchs, Switzerland) for His-tag fusion proteins. The concentration of each fusion protein was determined by Coomassie staining.

Isolation of nuclear and cytosolic proteins for tobacco tissue was performed with CELLYTPN1 CelLytic PN isolation/Extraction Kit (Sigma-Alsrich) according to the manufacturer’s instruction.

**Salt stress treatment**

To examine the effect of salt stress on transgenic *Arabidopsis* (*Arabidopsis thaliana*) plants, sterilized *Arabidopsis* seeds stratified for 2–3 days in darkness at 4°C were plated directly onto basal medium (half-strength MS medium, 3% sucrose) without (0 mM) or with 100 mM NaCl for germination rate tests and with 125 mM NaCl for testing salt sensitive phenotypes in-plate.

To examine the effect of salt stress on transgenic *Arabidopsis* plants, seeds were sown directly in soil. Seventeen-day-old pot-cultured plants were watered without (0 mM) or with 150 mM NaCl twice per week for 2 weeks to test their salt sensitive phenotypes in soil. To examine the effect of salt treatment on flowering time, *Arabidopsis* seeds were germinated on basal medium (1/2× MS, 3% sucrose) for 2–3 days and transferred to growth boxes (18 cm in height, four plants per box) containing basal medium without (0 mM) or with 50 mM NaCl.

To examine *PagGI* gene expression profiles, water was withheld from 2-month-old poplar plants cultured in 10 cm diameter pots for 2 days, followed by irrigation with water or 200 mM NaCl solution for 48 h. Various tissues including shoot tips (~1 cm from the top), young leaves (3^rd^–4^th^), mature leaves (15^th^), xylem (stems from 5^th^–15^th^ internode), bark (5^th^–15^th^ internode), and roots (10 cm length from root tip) were collected every 4 h. The samples were immediately frozen in liquid nitrogen and stored at –80°C.

To test the salt-tolerance of transgenic poplars *in vitro*, poplar stem cuttings with double nodes were cultured in RM supplemented with 0 (control), 50 mM, and 70 mM NaCl in tubes for 1 month. The root fresh weight of each individual plant was measured. Photographs were taken before and after the indicated treatment.

To examine the salt tolerance of transgenic poplars at the whole-plant level, water was withheld from 2-month-old poplar plantlets for 2 days, followed by irrigation with 200 mM NaCl solution every 3 d for 6 d, after which the plants were watered to allow them to recover from salt stress. At the indicated days of salt treatment, the plant height, shoot biomass, and root biomass of each individual plant were measured. Photographs were taken before and after treatment.

**Southern blot analysis**

Genomic DNA from poplar was extracted from leaves as described by Kim and Hamada (2005), separated on a 0.8% agarose gel after restriction digestion with *Eco*RI (Roche, Manheim, Germany), transferred to a Zeta-probe GT membrane (Bio-Rad, CA, USA), and hybridized with a ^32^P-labeled probe designed based on the *Bar* cDNA fragment.

**Figure S1** *PagGI* Genes are Involved in the Regulation of Circadian Rhythms. (a) Time course of *PagGIa*/*c* and *PagGIb* expression in mature poplar leaves under LDs. (b) Expression levels of *PagGIa*/*c* and *PagGIb* in different tissues at ZT12. Total RNA samples were collected every 4 h from 2-month-old poplar plants entrained in LDs of 48 h with or without 200 mM NaCl treatment. The mRNA abundance was quantified by quantitative RT-PCR, which was performed in triplicate with three independently harvested samples. *Actin* expression was used as an internal control. Shoot tip (S), (~1 cm from the top); Young leaf (Y), 3^rd^ to 5^th^ leaves; Mature leaf (M), 15^th^ leaf; Bark (B) and Xylem (X), 5^th^ to 15^th^ internode; Root (R), 10 cm length from root tip. White and black bars above the graph indicate day and night periods, respectively. Error bars represent SD of three independent experiments. Asterisks and ns indicate significant and nonsignificant differences at *P* < 0.05, respectively.

**Figure S2** Generation of Transgenic *Arabidopsis* Plants Overexpressing *PagGI* Genes (T3 Generation). (a) RT-PCR analysis of 35S:*PagGIa/b/c* *Col-0* (overexpression lines) and 35S:*PagGIa/b/c gi-2* (complemented lines) transgenic plants using *PagGI*-specific primers. The poplar *actin* gene was used as an internal control. (b) Immunoblotting analysis of transgenic plants. Samples were collected at ZT12 from 15-day-old plants cultured in MS basal medium. Equal amounts of plant protein were loaded. Immunoblot analysis with anti-GFP antibody and Coomassie brilliant blue (CBB)-stained blots are shown as a loading control. (c) Confocal microscope images of 35S:*PagGI* *Col-0* transgenic plants. The fluorescence was observed under a confocal laser-scanning microscope. Scale bars represent 50 µM.

**Figure S3** Sensitivity of Transgenic *Arabidopsis* Seedlings to Salt Stress. (a and b) Seed germination of WT, 35S:*PagGIa/b/c* *Col-0* (overexpression lines), and 35S:*PagGIa/b/c gi-2* (complemented lines) transgenic plants. Seeds harvested on the same day were plated on MS medium without (0 mM) or with 100 mM NaCl. Photographs were taken 15 d after planting, and the germination rate was determined for 7 d. Data represent the means ± SD of three independent experiments (n = 100). (c and d) Comparison of root growth in WT, *35S*:*PagGIa*/*b*/*c Col*-*0*, and *35S*:*PagGIa*/*b*/*c gi*-*2* plants. Seeds from the indicated lines were grown on MS basal medium without (0 mM) or with 125 mM NaCl under LDs, and photographed after 2 weeks. Relative fresh weight growth was measured at the end of the treatments. More than ten plants were measured per data point. Error bars represent the SD of three independent experiments. Asterisks and ns indicate significant and nonsignificant differences at *P* < 0.05, respectively.

**Figure S4** *SOS1*-*Like* Gene in Poplar. (a) Schematic of PagSOS1 depicting three domains: the N-terminal membrane domain (TM), the central domain sharing sequence homology with NHX8, and the C-terminal SOS1-specific domain. In the latter, the three dark gray boxes represent the domain essential for PagSOS1 activity (left), the auto-inhibitory domain (center), and the PagSOS2 phosphorylation site (right). (b) Hydropathicity plot of the deduced PagSOS1 and AtSOS1 amino acid sequences analyzed using the Kyte-Doolittle algorithm.

**Figure S5** *SOS2*-*Like* Gene in Poplar. (a) Schematic diagram of the domain structure of PagSOS2. (b) Amino acid sequence alignment of PagSOS2 and *Arabidopsis* SOS2 (AtSOS2). The alignment was performed using ClustalX 2.0 and DNAMAN software. Green frame indicates conserved regulatory domain between PagSOS2 and AtSOS2. (c) Subcellular localization of PagSOS2F- and PagSOS2C-GFP fusion proteins. Constructs containing PagSOS2F- and PagSOS2C-GFP were transiently overexpressed in *N. benthamiana* leaves by *Agrobacterium* infiltration. DAPI (second row) and GFP (third row) fluorescence was observed at 3 days after infiltration. Both fluorescence images are merged in the fourth row (merge). The fluorescence was observed under a confocal laser-scanning microscope. Scale bars represent 50 µM.

**Figure S6. Nuclear and cytosolic distribution of PagGI proteins.** Nuclear and cytosolic proteins from *N. benthamiana* transiently leaves co-expressing PagGI-GFP with PagSOS2TD-GFP. PagGIa, b, and c protein were detected via immunoblot analysis with anti-GFP antibody. Molecular weight markers in kDa.

**Figure S7** PagSOS2 Phosphorylates PagSOS1 *in vitro*. An *in vitro* kinase assay was performed including purified, bacterially produced GST-PagSOS1C (PagSOS1 C-terminus, amino acids 887–1146), GST-PagSOS2TD (Thr168 was changed to Asp in PagSOS2), and GST-PagSOS2 in the indicated combinations. Shown are an autoradiogram (top panel) and CBB stained (bottom panel) gel containing the resolved reaction products.

**Figure S8** Generation of Transgenic Poplar Plants by Down-Regulating *PagGI* Genes. (a) Genomic DNA PCR analysis of RB plants using InF/NotR and Bar gene-specific primers. P, DNA plasmid containing GI-S-Intron-GI-AS-Nos-ter and Bar fragment. (b) RT-PCR analysis of RG plants using PagGI-specific primers. The poplar *actin* gene was used as an internal control. (c) Southern blot analysis of RB plants. The integration and gene copy number of the construct in RB plants were confirmed by probing with a *Bar* gene fragment after *Xho*I digestion.

**Figure S9** Effects of Salt Stress on NT and RB Plants Grown under in-tube Conditions. (a) Root growth of NT and RB plants grown in RM supplemented with 0 (control), 50, and 70 mM NaCl in-tube for 1 month. (b) Root fresh weights of NT and RB plants at the end of the treatment (shown in A) were measured. More than three plants were measured per data point. Error bars represent SD of three independent experiments. Asterisks and ns indicate significant and nonsignificant differences at *P* < 0.05, respectively.

**Figure S10** *ZEITLUPE* (ZTL)-*Like* Genes in Poplar. (a) Amino acid sequence alignment of PagZTL1/2 and *Arabidopsis* ZTL (AtZTL). The alignment was performed using ClustalX 2.0 and DNAMAN software. The PAS superfamily/LOV domain and other signature motifs are indicated with solid lines of different colors. (b) Phylogenetic analysis of poplar *ZTL-like* genes. Phylogenetic tree of ZTL proteins from poplar and other species constructed with MEGA 6.06 software. Scale bar indicates 0.05 substitutions per amino acid site. Full-length amino acid sequences were aligned, and bootstrap analysis was performed based on 1000 replicates. (c) Subcellular localization of PagZTL1- and 2-GFP fusion proteins. Constructs containing PagZTL1- and 2-GFP were transiently overexpressed in *N. benthamiana* leaves by *Agrobacterium* infiltration. DAPI (second row) and GFP (third row) fluorescence was observed at 3 days after infiltration. Both fluorescence images are merged in the fourth row (merge). The fluorescence was observed under a confocal laser-scanning microscope. Scale bars represent 50 µM.

**Figure S11** The Interaction Between PagGI Proteins and PagZTL Proteins *in vivo*. Constructs containing Venus fluorescent protein N-terminal (VN) alone or tagged with PagGIa, b, and c (PagGIa-, b- and c-VN), and constructs containing Venus fluorescent protein C-terminal (VC) alone or tagged with PagZTL1 and 2 (VC-PagZTL1 and 2) in the indicated combinations, were transiently overexpressed in *N. benthamiana* leaves by *Agrobacterium* infiltration. DAPI (second row) and Venus (third row) fluorescence was observed at 3 days after infiltration. Both fluorescence images are merged in the fourth row (merge). The fluorescence was observed under a confocal laser-scanning microscope. Scale bars represent 50 µM.

**Figure S12** *CONSTANS* (*CO*)-*Like* Gene in Poplar. (a) Amino acid sequence alignment of PagCO2 and *Arabidopsis* CO (AtCO). The alignment was performed using ClustalX 2.0 and DNAMAN software. The zinc finger, B-box domain, and other signature motifs are indicated by solid lines of different colors. (b) Subcellular localization of PagCO2-GFP fusion protein. Constructs containing PagCO2-GFP were transiently overexpressed in *N. benthamiana* leaves by *Agrobacterium* infiltration. DAPI (second row) and GFP (third row) fluorescence was observed at 3 days after infiltration. Both fluorescence images are merged in the fourth row (merge). The fluorescence was observed under a confocal laser-scanning microscope. Scale bars represent 50 µM.

**Figure S13** The Interaction Between PagGI Proteins and PagCO2 Protein *in vivo*. Constructs containing Venus fluorescent protein N-terminal (VN) alone or tagged with PagGIa, b, and c (PagGIa-, b- and c-VN), and constructs containing Venus fluorescent protein C-terminal (VC) alone or tagged with PagCO2 (VC-PagCO2) in the indicated combinations, were transiently overexpressed in *N. benthamiana* leaves by *Agrobacterium* infiltration. DAPI (second row) and Venus (third row) fluorescence was observed at 3 days after infiltration. Both fluorescence images are merged in the fourth row (merge). The fluorescence was observed under a confocal laser-scanning microscope. Scale bars represent 50 µM.

**Table S1.** Gene specific primers used in this study.

| Primer name | Sequence (5'-...- 3') | Application |
| --- | --- | --- |
| *PagGI*-F | ATGGCTAGTTCTTCTTCTG | Gene cloning of PagGIa, b and c |
| *PagGI*-R | TCAAATGGAAATAGTACAGCC | Gene cloning of PagGIa, b and c |
| *PagZTL1*-F | ATGGAGTGGGATAGCAATTCGG | Gene cloning of PagZTL1 |
| *PagZTL1*-R | TCAGATAACTGAACTAGCCAAG | Gene cloning of PagZTL1 |
| *PagZTL2*-F | ATGGAGTGGGATAGCGATTCGG | Gene cloning of PagZTL2 |
| *PagZTL2*-R | TTAGGTGGTTGAACTTCCCAAAG | Gene cloning of PagZTL2 |
| *PagCO2*-F | ATGTTGAAGCAAGAGAGTAGTG | Gene cloning of PagCO2 |
| *PagCO2*-R | TCAGAATGATGGGACAATGCC | Gene cloning of PagCO2 |
| *PagSOS1*-F | ATGGGGAGTGCGATAGAAAAAG | Gene cloning of PagSOS1 |
| *PagSOS1*-R | CTAAGAAGCATGATGGAACG | Gene cloning of PagSOS1 |
| *PagSOS2*-F | ATGATGAAGAAAGTAACGAG | Gene cloning of PagSOS2 |
| *PagSOS2*-R | TCATTGTTCGATGCAGGCAGG | Gene cloning of PagSOS2 |
| *attB1 adapter* | GGGGACAAGTTTGTACAAAAAAGCAGGCT | Gateway |
| *attB2 adapter* | GGGGACCACTTTGTACAAGAAAGCTGGGT | Gateway |
| *PagGI*-GW-F | AAAAAGCAGGCTTCATGGCTAGTTCTTCTTCTG | Plasmid construction for subcellular localization (SL), BIFC, and pull-down |
| *PagGI*-GW-R | AGAAAGCTGGGTCAATGGAAATAGTACAGCC | Plasmid construction for SL, BIFC, and pull-down |
| *PagZTL1*-GW-F | AAAAAGCAGGCTTCATGGAGTGGGATAGCAATTCGG | Plasmid construction for SL and BIFC |
| *PagZTL1*-GW-R | AGAAAGCTGGGTCGATAACTGAACTAGCCAAG | Plasmid construction for SL and BIFC |
| *PagZTL2*-GW-F | AAAAAGCAGGCTTCATGGAGTGGGATAGCGATTCGG | Plasmid construction for SL and BIFC |
| *PagZTL2*-GW-R | AGAAAGCTGGGTCGGTGGTTGAACTTCCCAAAG | Plasmid construction for SL and BIFC |
| *PagCO2*-GW-F | AAAAAGCAGGCTTCATGTTGAAGCAAGAGAGTAGTG | Plasmid construction for SL and BIFC |
| *PagCO2*-GW-R | AGAAAGCTGGGTCGAATGATGGGACAATGCC | Plasmid construction for SL and BIFC |
| *PagSOS1F*-GW-F | AAAAAGCAGGCTTCATGGGGAGTGCGATAGAAAAAG | Plasmid construction for SL |
| *PagSOS1F*-GW-R | AGAAAGCTGGGTCAGAAGCATGATGGAACGAAAGC | Plasmid construction for SL |
| *PagSOS1C*-GW-F | AAAAAGCAGGCTTCAGGTCAGTGATGACCACATACATC | Plasmid construction for kinase assay |
| *PagSOS2F*-GW-F | AAAAAGCAGGCTTCATGATGAAGAAAGTAACGAG | Plasmid construction for SL, pull-down and kinase assay |
| *PagSOS2F*-GW-R | AGAAAGCTGGGTCTTGTTCGATGCAGGCAGG | Plasmid construction for SL, pull-down and kinase assay |
| *PagSOS2C*-GW-F | AAAAAGCAGGCTTCGTAATGAATGCATTTGAGATG | Plasmid construction for SL, pull-down and kinase assay |
| *SOS2T168D*-F | CAGAAAGGGGTTGGACTTCTTCATGATACATGTGGA | Plasmid construction for constitutively active form of PagSOS2 |
| *SOS2T168D*-R | AACATAATTCGGGGTTCCACATGTATCATGAAGAAG | Plasmid construction for constitutively active form of PagSOS2 |
| *SOS1*-IF-F | TCGACCCAGCCTCGAGATGGGGAGTGCGATAGAAAAAGG | Yeast experiments |
| *SOS1*-IF-R | CGCGCGGCCGCTCGAGAGAAGCATGATGGAACGAAAGC | Yeast experiments |
| *SOS2*-IF(S-E)-F | TACAATCAACTCCCCGGGATGATGAAGAAAGTAACGAG | Yeast experiments |
| *SOS2*-IF(S-E)-R | TAGAAGTGTCGAATTCTTGTTCGATGCAGGCAGGAAT | Yeast experiments |
| *GI*-IF(B-S)-F | ATCAAAGCTGCCTAGGATGGCTAGTTCTTCTTCTGAG | Yeast experiments |
| *GI*-IF(B-S)-R | TACGGACGTCCAGCTGAATGGAAATAGTACAGCCTAAC | Yeast experiments |
| *35S*-F | CTTGCATGCCTCGAGACTAGAGCCAAGCTGATCTCC | Plasmid construction for RNAi |
| *35S*-R | TCGACTAGAATAGTAAATTGTAATG | Plasmid construction for RNAi |
| GI-S-F | TACTATTCTAGTCGAAGCGCATCAGATCTACTTCTC | Plasmid construction for RNAi |
| GI-S-R | CCGCCACCGCGGTGGAGCTCAGAAAGGCATCGTATAGTCGC | Plasmid construction for RNAi |
| Intron-F | CCGGGTACCGAGCTCAAATTTCTAGTTTTTCTCCTTCAT | Plasmid construction for RNAi and RT-PCR |
| Intron-R | TTCTGTAACTATCATCATCATCAT | Plasmid construction for RNAi |
| GI-AS-F | ATGATAGTTACAGAAAGAAAGGCATCGTATAGTCGC | Plasmid construction for RNAi |
| GI-AS-R | TCAATCTTAAGAAACAGCGCATCAGATCTACTTCTC | Plasmid construction for RNAi |
| Nos-F | GTTTCTTAAGATTGAATCCTGTT | Plasmid construction for RNAi |
| Nos-R | CGGGGGATCCACTAGTCCCGATCTAGTAACATAGATG | Plasmid construction for RNAi and RT-PCR |
| *PagGIs*-F | GGAGCAAGAGACCAAGAGGAGAAG | Real-time PCR |
| *PagGIs*-R | CAGAGCACATACAGCAGCCAGAA | Real-time PCR |
| *PagGIa/c*-F | TGGACATGCTGTTGTTCTTCCT | Real-time PCR |
| *PagGIa/c*-R | GATTCACTACTTGTAGCCTGGCTG | Real-time PCR |
| *PagGIb*-F | TGGACATGCTGTTGTTCATCCA | Real-time PCR |
| *PagGIb*-R | CTGATTCAATACTTGTAGAATGGCCA | Real-time PCR |
| *IAA1*-F | ATCATGAAAGGGTCTGAGGCCA | Real-time PCR |
| *IAA1*-R | TTCCAGGCTTCAAAGCTCGATG | Real-time PCR |
| *IAA2*-F | CCAGCTGTTCGCATGAATGTTG | Real-time PCR |
| *IAA2*-R | CCCAAATGCAGGTCTTTGGAGA | Real-time PCR |
| *IAA4*-F | AAGGAGGGGGATTGGTTGATTG | Real-time PCR |
| *IAA4*-R | TCAAGGAGAAAAGGAGACCGCA | Real-time PCR |
| *IAA5*-F | ATCATGAGGATGTCCGAGGCAA | Real-time PCR |
| *IAA5*-R | AATCCAACACAAAGCCGCTGA | Real-time PCR |
| *Actin*-F | GCCATCTCTCATCGGAATGGAA | RT-PCR and Real-time PCR |
| *Actin*-R | AGGGCAGTGATTTCCTTGCTCA | RT-PCR and Real-time PCR |
| *Bar*-F | TGGGTTGTTGTAAAGGACGACTCA | Southern blot |
| *Bar*-R | GCCACCTAATTCAGCGACTTCTG | Southern blot |

**Table S2.** GenBank accession number used in this study.

| Gene name | GenBank accession number |
| --- | --- |
| *PagGIa* | KT875324 |
| *PagGIb* | KT875325 |
| *PagGIc* | KT875326 |
| *PagZTL1* | KT875327 |
| *PagZTL2* | KT875328 |
| *PagCO2* | KT875329 |
| *PagSOS1* | KT875330 |
| *PagSOS2* | KT875331 |
